# 6-Bromoindirubin-3′-oxime intercepts GSK3 signaling to promote and enhance skeletal muscle differentiation affecting miR-206 expression in mice

**DOI:** 10.1038/s41598-019-54574-4

**Published:** 2019-12-02

**Authors:** Elvira Ragozzino, Mariarita Brancaccio, Antonella Di Costanzo, Francesco Scalabrì, Gennaro Andolfi, Luca G. Wanderlingh, Eduardo J. Patriarca, Gabriella Minchiotti, Sergio Altamura, Francesca Varrone, Vincenzo Summa

**Affiliations:** 1 IRBM S.p.A, 80131 Naples, Italy; 2 Department of Biology and Evolution of Marine Organisms, Stazione Zoologica Anton Dohrn, Naples, Italy; 3 Telethon Institute of Genetics and Medicine (TIGEM), 80078 Pozzuoli (NA), Italy; 4 Stem Cell Fate Laboratory, Institute of Genetics and Biophysics, ‘A. Buzzati-Traverso’, CNR, 80131 Naples, Italy; 5 IRBM S.p.A, 00071 Pomezia, Italy; 6 Department of Pharmacy, University of Naples “Federico II”, 80131 Naples, Italy

**Keywords:** Cell biology, Drug discovery

## Abstract

Dystrophies are characterized by progressive skeletal muscle degeneration and weakness as consequence of their molecular abnormalities. Thus, new drugs for restoring skeletal muscle deterioration are critically needed. To identify new and alternative compounds with a functional role in skeletal muscle myogenesis, we screened a library of pharmacologically active compounds and selected the small molecule 6-bromoindirubin-3′-oxime (BIO) as an inhibitor of myoblast proliferation. Using C2C12 cells, we examined BIO’s effect during myoblast proliferation and differentiation showing that BIO treatment promotes transition from cell proliferation to myogenic differentiation through the arrest of cell cycle. Here, we show that BIO is able to promote myogenic differentiation in damaged myotubes *in-vitro* by enriching the population of newly formed skeletal muscle myotubes. Moreover, *in-vivo* experiments in CTX-damaged TA muscle confirmed the pro-differentiation capability of BIO as shown by the increasing of the percentage of myofibers with centralized nuclei as well as by the increasing of myofibers number. Additionally, we have identified a strong correlation of miR-206 with BIO treatment both *in-vitro* and *in-vivo*: the enhanced expression of miR-206 was observed *in-vitro* in BIO-treated proliferating myoblasts, miR-206 restored expression was observed in a forced miR-206 silencing conditions antagomiR-mediated upon BIO treatment, and *in-vivo* in CTX-injured muscles miR-206 enhanced expression was observed upon BIO treatment. Taken together, our results highlight the capacity of BIO to act as a positive modulator of skeletal muscle differentiation *in-vitro* and *in-vivo* opening up a new perspective for novel therapeutic targets to correct skeletal muscle defects.

## Introduction

Skeletal muscle is the largest tissue mass of the body voluntarily controlled by the organism. During embryonic myogenesis, mesoderm-derived structures generate bundles of multinucleated myofibers. Proliferation and differentiation phases lead to the multinucleated myofibers formation: during perinatal phase, muscle resident myogenic progenitors (myoblasts) initially proliferate extensively but later, during the differentiation phase, they start to fuse and form multinucleated myotubes leading to the creation of myofibers^1–3^. Commitment to terminal differentiation is a finely regulated multi-stage process which initiates with the exit of myoblast from cell cycle, expression of myotube-specific gene, cell fusion to form multinucleated myotubes and the maturation of myotubes into myofibers^4,5^. *In-vitro* cultured C2C12 cell line is a widely used model to study several aspects of skeletal myogenesis. The C2C12 cells are myoblast cells derived from mouse satellite cells. They readily proliferate in high-serum conditions while differentiate into multinucleated myotubes following withdrawal of serum or mitogens from myoblast cultures. The morphology of C2C12 cells change from flat, fusiform or star-shaped mono-nucleated cells into fused multinucleated MHC-positive cells^6–8^. Since myogenic differentiation is an essential part of skeletal muscle growth finely regulated by the expression of stage-specific markers, including MyoD, Myogenin and MHC. The most widely accepted method to measure the progression of skeletal muscle differentiation is represented by the calculation of Fusion Index that measures the amount of the fused skeletal muscle cells^10^. Several intracellular signaling pathways are involved in myogenic differentiation, including p38 MAPK, ERK/MAPK, PI3K/AKT and Wnt signaling^9,11^. A component in Wnt signaling, Glycogen synthase kinase 3 (GSK3), a kinase of Wnt pathway, has been proposed as key regulator of skeletal muscle differentiation^12^ and associated with the regulation of muscle mass: GSK3 is required for the induction of muscle atrophy *in-vitro*^13^ and its absence accelerates muscle mass recovery and post-natal myogenesis of atrophied muscle^14,15^. GSK3 inhibition also has been shown to have a positive effect on myogenesis in myotonic dystrophy mouse model^16^ and in Myotonic Dystrophy 1 (DM1) patients currently enrolled in GSK3-based clinical trials of L.Timchenko. Additionally, studies have shown that inactivation of GSK3 is also able to stimulate muscle oxidative metabolism and protects against disuse-induced loss of oxidative gene expression in skeletal muscle^17^. Importantly, GSK3 inhibitors, including Lithium Chloride (LiCl), CHIR (CHIR99021)^18^ and 6-bromoindirubin 3′-oxime (BIO), have been shown to mimic the activation of Wnt signaling^19–21^ and potentially increase muscle differentiation.

LiCl maintains growth proliferation and survival of C2C12 cells, while no evidence of BIO’s effect on C2C12 cell proliferation has been reported yet. Additionally, BIO and CHIR have shown potent impact on ESC self-renewal and have proven useful for *in-vitro* mesoderm differentiation^22^. Muscle differentiation is a complex process also regulated by a set of muscle-specific microRNAs^23^ that belongs to the myomiR family (miR-133a, miR-133b, miR-206, miR-208a, miR-208b and miR-499). In particular, it has been revealed that the overexpression of miR-206 in C2C12 cells is able to block cell cycle progression and to induce myotubes formation, whereas the inhibition of miR-206 expression produces the opposite effect^24^. However, the specific role of Wnt pathway signaling activation in myomiRs regulations needs to be further clarified. Here, our findings demonstrate that BIO is able to enhance miR-206 expression and to improve myogenic differentiation in both healthy and damaged skeletal muscle fibers *in-vitro*. Moreover, our *in-vivo* studies also highlight a new potential role of BIO in the regeneration process of the injured TA muscles.

## Materials and Methods

### Compounds

The LOPAC®1280 library, consisting of 1280 pharmacologically active compounds, 6-bromoindirubin-3′-oxime (BIO) and cobra snake venom cardiotoxin (CTX) were purchased from Sigma.

### Cell line and AntagomiR-206 transfection

Mouse C2C12 cells were obtained from ATCC and cultured in the following media: Growth Medium (GM) containing Dulbecco’s Modified Eagle Medium (DMEM; Gibco) supplemented with 10% Fetal Bovine Serum (FBS; Gibco), 1% glutamine and 1% antibiotics (100 U/ml Penicillin and 100 μg/ml Streptomycin; Gibco); Differentiation Medium (DM) containing DMEM supplemented with 2% adult Horse Serum (Gibco), 1% glutamine and 1% antibiotics (100 U/ml Penicillin and 100 μg/ml Streptomycin; Gibco). C2C12 cells were seeded in 6-well plate format (2.5 × 10^5^ cells/well) in GM medium for 16 hours and then transfected with 50 nM of AntagomiR-206 and negative control (Exiqon) using Lipofectamine 2000 (Invitrogen) method according to the manufacturer’s protocol. Cells were treated with GM, DM, BIO (3 µM in GM medium) or Vehicle (DMSO) for 24 h. The same experiment was performed and cells were treated with GM, DM, CHIR (3 µM in GM medium) or Vehicle (DMSO) for 24 h.

### Proliferation and viability assays

C2C12 cells, plated in 96-well plates (5 × 10^3^ cells/well) were incubated with GM, DM, BIO (3 μM dissolved in GM medium) or Vehicle (DMSO) for 24 h and 48 h. The same experiment was performed and C2C12 cells were incubated with GM, DM, CHIR (3 μM dissolved in GM medium) or Vehicle (DMSO) for 24 h and 48 h. Cell proliferation was measured by CellTiter-Glo® Luminescent Cell Viability Assay (G7570, Promega) using the microplate reader DTX880 Multimode Detector (Beckman Coulter). CellTox™ Green Cytotoxicity Assay (G8741, Promega) was used to determine toxic effects during or after long-term exposure of cells in culture. BIO compound was tested in triplicate on n = 5 independent experiments. Data were expressed as a percentage of GM treated cells.

### Quantitative real-time PCR

RNA was extracted from cultured cells using TRIzol reagent (Ambion) followed by isopropanol-alcohol precipitation (RNeasy Mini Kit, Qiagen) before quantitation. RNA was then converted to cDNA with High Capacity cDNA Reverse Transcription kit (Applied Biosystem) according to the manufacturer’s protocol. Gene expression analysis was carried out using GAPDH or β-Tubulin reference gene. Primers for mouse CyclinD1 (CycD1), Myogenin (Myog), mouse myogenic differentiation 1 (MyoD1), Pax7, Follistatin (FST), Myosin Heavy Chain 2 (Myh2), β-Catenin, Calcineurin, Utrophin, Transforming growth factor beta (TGF-β), Cullin-1, MRF4, MuRF-2, FBXW7 and β-TrCP are reported in Supplementary Table S1. Transcript levels were assessed using the Bio-Rad CFX machine according to the manufacturer’s instructions and each experiment was repeated three times using independent RNA samples. For microRNAs quantitative reverse transcription–polymerase chain reaction, primers for mature miR-206, miR-133, miR-27b, the internal control U6, and miR-16 were used according to the manufacturer’s protocol (MiRCURY LNA microRNA system control primer set, Exiqon, Vedbaek, Denmark).

### Immunoblotting, immunofluorescence and antibodies

C2C12 cells, plated in 10 cm plates (8 × 10^5^ cells/well) were incubated with GM, BIO (3 μM dissolved in GM medium) or Vehicle (DMSO) for 24 h and 48 h. Total cell lysates were obtained with RIPA buffer supplemented with fresh 1X protease inhibitors (Complete Ultra Tablets Mini easypack, Roche) and phosphatase inhibitor cocktail tablets (phos STOP easypack, Roche). Commercial antibodies used in this study include anti-GSK3 (Cell Signaling), anti-GAPDH (Abcam), anti-Cyclin D1 (Santa Cruz) and anti-β-Catenin (Abcam). For immunofluorescence experiments, C2C12 cells were plated in 96-well plates (5 × 10^3^ cells/well). After treatments, cells were washed with 1X PBS (Phosphate Buffer Saline, Gibco) and then fixed with 4% paraformaldehyde for 10 min. After three washes in 1X PBS, cells were permeabilized with 0.5% Triton X-100 for 10 min and washed with 1X PBS. Cells were then blocked with 20% goat serum in 1X PBS (Gibco) for 30 min and then incubated overnight at 4 °C with anti-MHC antibody (Myosin 4 Monoclonal Antibody MF20, Alexa Fluor 488, eBioscience™). Cells were then washed three times with 1X PBS and subjected to DAPI staining (Life Technologies). Cells were examined with the DMI600 (Leica) fluorescence microscope and photographed using a DFC360-FX camera (Leica). Images were analyzed with ImageJ software to select MHC-positive cells in order to discriminate between cell populations (mononucleated, binucleated, <5 nuclei, >5 nuclei cells). Fusion Index was used to evaluate BIO effect on cell population. DMSO was used as negative control (Vehicle). Each experiment was performed in triplicate.

### Nuclear protein extraction, immunoblotting and antibodies

C2C12 cells, plated in 10 cm plates (8 × 105 cells/well) were incubated with GM, BIO (3 μM dissolved in GM medium) or Vehicle (DMSO) for 24 h and 48 h. To obtain the differential protein extraction (nuclei/cytoplasm) the cells were washed with cold 1X PBS (Gibco) and pelleted by centrifugation at 200 g for 7 minutes. All samples were suspended in appropriate quantity of STM buffer (250 mM sucrose, 50 mM Tris-HCl pH 7.4, 5 mM MgCl2, protease and phosphatase inhibitor cocktails), maintained on ice for 30 minutes, vortexed for 15 seconds and centrifuged at 800 g for 15 minutes. The pellet was resuspended in an adequate volume of STM buffer, vortexed for 15 seconds and centrifuged at 500 g for 15 minutes. The pellet was resuspended in adequate volume of NET buffer (20 mM HEPES pH 7.9, 1.5 mM MgCl2, 0.5 M NaCl, 0.2 mM EDTA, 20% glycerol, 1% Triton-X-100, protease and phosphatase inhibitor cocktails) and vortexed for 15 seconds prior to incubation on ice for 30 minutes. The nuclei were lysed with 10–20 passages through an 18-gauge needle and the lysate was centrifuged at 9000 g for 30 minutes. The resultant supernatant was the final nuclear fraction. Western blot was performed and commercial antibodies used in this study include anti-β-Catenin (Abcam) and anti-Histone H3 (Abcam).

### Mouse skeletal myoblasts and myotubes treatments and analysis

To assess the effect of BIO a on mouse skeletal myoblasts differentiation, C2C12 cells (70% confluency) were treated 24 h with GM, DM, BIO 3 µM in GM medium or Vehicle (DMSO) and then incubated in GM or DM for 96 h. The fusion index was calculated as the ratio of the number of nuclei inside myotubes (MHC-positive cells) to the number of total nuclei at day 4 of myogenic differentiation. The number of nuclei was estimated by the average of nuclei counted in 15 independent and randomly chosen microscope fields (10×) in order to measure the size of the resulting myotubes defined by the presence of at least three nuclei within a continuous cell membrane. Data are expressed as mean diameter in micrometers of ten myotubes measured per field. The average size (diameter) per myotube was calculated as the mean of three measurements taken along the long axis of the myotube. To assess the effect of BIO on myotubes after damage, C2C12 cells (70% confluency) were treated with DM for 96 h, then the damage was performed by 1 μM Cardiotoxin (CTX) (Sigma) treatment for 24 h. Myotubes CTX damaged were treated with GM, DM, BIO 3 µM dissolved in GM medium or Vehicle (DMSO) for 24 h.

### Fluorescence-activated cell sorting analysis

Cell cycle distribution was determined by measuring the amount of cellular DNA using Propidium Iodide (PI; Sigma) staining. Cells were collected by centrifugation and fixed with 70% ethanol (4 °C, 1 h). Cells were then washed with 1X PBS, treated with RNase A (Sigma) for 1 h, and then incubated with 20 μg/ml PI for additional 30 min. The DNA content was determined using a flow cytometer (Canto II, BD) by measuring PI emission at 580 nm. Cell-cycle distribution was analyzed using BD FACS Diva software.

### Animals

8-week-old wild-type C57Bl/6 J mice were used in this study. The TA muscle was injured by injection of 10 μl cardiotoxin (10 μM, Sigma, St. Louis, MO, USA). The cardiotoxin (CTX) injury model is widely used to examine skeletal muscle regeneration. The injection of CTX into rodent muscle induces inflammation and myofiber degeneration that is followed by a regenerative response. Treatment: (2′Z,3′E)-6-Bromoindirubin-3′-oxime (BIO) was reconstituted in 1X PBS at a concentration of 5 mM (B1686, Sigma) combined with saline as Vehicle and injected directly into the TA muscle in a volume of 10 μl at a final concentration of 1 μM. All animal studies were performed in accordance with approved protocols by the Institute of Genetics and Biophysics, ‘A. Buzzati-Traverso’, CNR and were conducted according to EU Directive 2010/63/EU for animal experiments.

### RNA preparation from TA muscle

Total RNAs from the TA muscle were isolated using RNeasy mini kit (Qiagen) according to the manufacturer’s instruction. 1μg of total RNA was utilized for cDNA synthesis using reverse transcriptase (Qiagen) and random hexamers. QRT-PCR assay was performed using SYBR Green PCR master mix (Qiagen).

### TA muscle sections and immunostaining

Muscles were freshly frozen and cut in cryostat sections. Slides were fixed in 4% (wt/vol) paraformaldehyde (PFA), permeabilized with 0.5% Triton X-100 (Sigma–Aldrich). Primary antibodies used are as follows: laminin (1:200; Sigma), F4/80 (1:100; Serotec), and MHC (1:200, thermofisher). Appropriate fluorophore-conjugated secondary antibodies (Alexa Fluor 488) were used. Vectashield medium containing DAPI (Vector Laboratories) was used for mounting. Sections incubated without primary antibodies served as controls. Labeling was visualized by epifluorescent illumination using an Axiovision microscope (Carl Zeiss). The number of myofibers with central nuclei, the number of total myofibers and CSA (cross sectional area) of fibers were morphometrically analyzed with ImageJ software.

### Statistical analyses

All statistical analyses were performed using the GraphPad Prism 5.1 software (GraphPad Software Inc., La Jolla, USA). Data were expressed as the mean ± standard deviation. A one-way analysis of variance (ANOVA) with Dunnett’s post hoc test was performed for multiple comparisons in proliferation and viability assays. A one-way analysis of variance (ANOVA) was performed for multiple comparisons in gene expression, fusion index, myotube size and proliferation assay. A two-way ANOVA was performed for multiple comparisons in myofibers number, CSA and gene expression in *in-vivo* experiments. Unpaired two-tailed Student’s t-test was performed for comparisons between uninjured and injured mice *in –vivo* and for gene expression data in Supplementary Information. Expression values of genes of interest were calculated on ΔΔCt type of analysis normalizing on GAPDH, β-Tubulin or U6 snRNA reference genes.

## Results

### Identification of BIO as inhibitor of myoblasts proliferation

**Figure 1:**
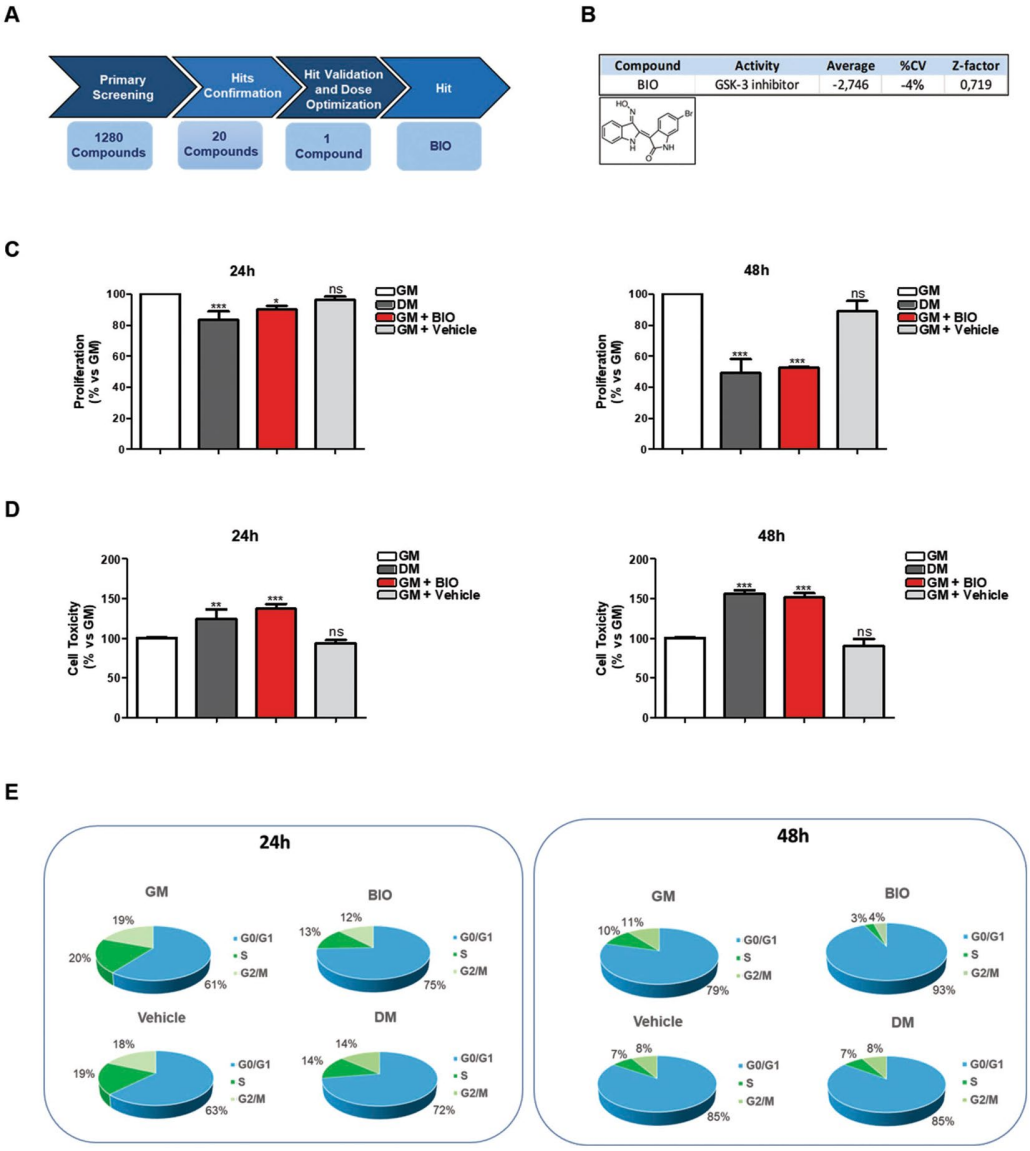
Identification of BIO as inhibitor of myoblasts proliferation. (**A**) Schematic representation of the screening cascade: C2C12 cells were probed with the low-molecular weight LOPAC®1280 library, consisting of 1280 pharmacologically active compounds. Identification of BIO as molecule affecting cell proliferation. (**B**) Chemical structure of BIO compound. (**C**) C2C12 cells were cultured in the presence of growth medium (GM), differentiation medium (DM), BIO (3 μM) or Vehicle (DMSO), for 24 h and 48 h. The effect of BIO on both proliferation and viability of C2C12 cells was measured by CellTiter-Glo® Assay and (**D**) by CellTox™ Green Cytotoxicity Assay, respectively. Data were collected from three independent experiments and represent means ± SEM relative to untreated cells (according to one-way analysis of variance ANOVA using the Dunnett’s Multiple Comparison Test). (**E**) C2C12 cells were treated as in C and D panels and cell-cycle distribution was analyzed by flow cytometry. Data (collected from four independent experiments) represent means ± SEM of cells in each phase of the cell cycle compared to a no-treatment group.

Myogenesis is a tightly regulated process. Embryonic precursors must be committed to myogenic lineage and the immature myoblasts have to exit from the cell cycle. To identify small molecules able to reduce the proliferation of C2C12 cells, a well-established *in-vitro* model of muscle differentiation^7,10^, we developed a cell-based assay using the LOPAC®1280 library. The LOPAC®1280 library, consisting of 1280 pharmacologically active compounds, was screened in a 96-well format at a final concentration of 12.5 μM on C2C12 cells under normal growth conditions. The screening cascade is represented in Fig. 1A. Our screening identified a total of 20 hit-compounds that significantly inhibited C2C12 proliferation. As negative control, we used DMSO (Vehicle) at a final concentration of 0.5% (the final concentration employed to dissolve BIO) that does not affect significantly C2C12 viability and toxicity (data not shown). Among them, we focused our attention on 6-bromoindirubin-3′oxime (BIO), a well-known GSK3 inhibitor that also acts as an agonist of the Wnt-β-catenin signaling pathway (Fig. 1B). Muscle differentiation is usually induced by growing C2C12 cells up to 70% confluence in Growth Medium (GM) and then by shifting the cells to Differentiation Medium (DM) consisting of DMEM plus adult Horse Serum (HS). Moreover, it is known that early, during muscle differentiation, a reduction of cell proliferation occurs. To validate the screening results, BIO was dissolved in GM medium and its activity was examined in comparison with GM, DM or Vehicle (DMSO) for 24 and 48 h by Cell Titer-Glo and Cell Toxicity assays performed on C2C12 cells to evaluate cell proliferation and viability upon BIO treatment. The proliferation capability of BIO-treated C2C12 cells was reduced at 24 h with a clear effect at 48 h (Fig. 1C). A slightly effect on C2C12 toxicity, comparable to DM-treated cells was occurred (Fig. 1D). Furthermore, the effect of BIO on proliferation and cell toxicity evaluated at 24 and 48 h revealed the presence of lower number of myoblasts indicating an apparent impairment of proliferation as well as shown in DM treatment when compared to the control cells (GM). These observations show that BIO treatment is able to inhibit C2C12 proliferation efficiently as DM. Experiments in the same conditions were also performed to investigate changes in the nuclei number amount showing that BIO is able to decrease nuclei number in myoblasts confirming its role on cell proliferation inhibition (Supplementary Information, Fig. S1A). Cell cycle arrest is a prerequisite for myoblast fusion and subsequent differentiation, so proliferation and differentiation of skeletal myoblasts are mutually exclusive events. To better investigate into the effect of BIO on myoblasts cell cycle progression, sub-confluent and asynchronous C2C12 cells were cultured in GM as proliferating myoblasts. The differentiation of myoblasts into myotubes was induced replacing GM conditions (10% of Fetal Bovine Serum) with low serum DM medium (2% of HS). Cells were incubated for 24 and 48 h in GM or DM conditions with or without BIO dissolved in GM medium (3 μM) and cell cycle was analyzed by flow cytometry. We observed that C2C12-BIO treated cells were arrested in G0/G1 phase of cell cycle unlike control cells (DMSO) in a time-dependent manner. BIO treatment (24 h) increased the fraction of cells in the G0/G1 phase from 63% of total in DMSO-treated cells (Vehicle) to either 75% in BIO or 72% in DM-treated cells (Fig. 1E left panel). The fraction of cells in the G0/G1 phase further increased (up to 93%) after 48 h of incubation (Fig. 1E right panel). Moreover, C2C12-BIO treated cells show a reduction in S phase. Taken together, our data revealed that BIO treatment impairs myoblasts proliferation promoting C2C12 cell cycle arrest in G0/G1 phase.

### BIO impairs myoblasts cell-cycle progression affecting the expression of cell cycle markers and myomiRs *in-vitro*

**Figure 2:**
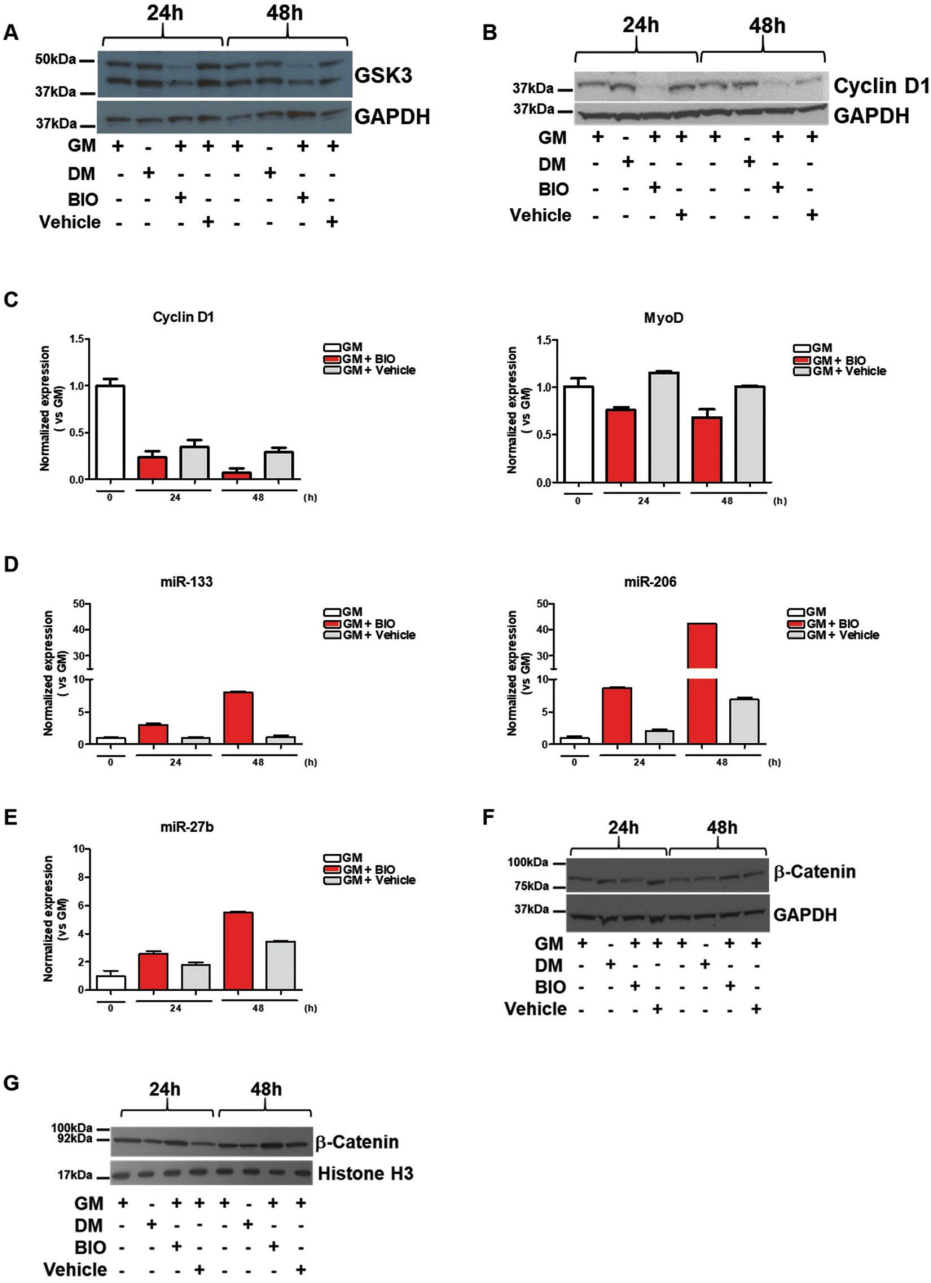
BIO impairs myoblasts cell-cycle progression affecting the expression of cell cycle markers and myomiRs *in-vitro*. C2C12 cells were cultured in the presence of growth medium (GM), differentiation medium (DM), BIO (3 μM) or Vehicle (DMSO), for 24 h and 48 h. (**A,B**) Cell lysates were subjected to Western blot analysis of GSK3β, Cyclin D1, and GAPDH protein expression. (**C**) qRT-PCR analysis of Cyclin D1 and MyoD in C2C12 treated with BIO (3 μM) or Vehicle (DMSO), for 24 h and 48 h. Data (collected from three independent experiments) represent means ± SEM relative to Vehicle treated cells. Data were analyzed by two-way analysis of variance ANOVA for Cyclin D1 (***P < 0.001) and for MyoD (*P < 0.05). (**D**) qRT-PCR analysis of miR-133 and miR-206 expression in C2C12 upon BIO (3 μM) or Vehicle (DMSO) treatment for 24 h and 48 h. Data (collected from three independent experiments) represent means ± SEM. Data were analyzed by two-way analysis of variance ANOVA (***P < 0.001). (**E**) qRT-PCR analysis of miR-27b expression in C2C12 upon BIO (3 μM) or Vehicle (DMSO), treatment for 24 h and 48 h. Data (collected from three independent experiments) represent means ± SEM. Data were analyzed by two-way analysis of variance ANOVA (*P < 0.05). (**F**) Cell lysates were subjected to Western blot analysis of β-Catenin and GAPDH expression in C2C12 upon GM, DM, BIO (3 μM) or Vehicle (DMSO), treatment for 24 h and 48 h. Data were collected from three independent experiments. (**G**) Cell lysates were subjected to Western blot analysis of nuclear β-Catenin and H3 expression in C2C12 upon GM, DM, BIO (3 μM) or Vehicle (DMSO), treatment for 24 h and 48 h.

In order to investigate the role of BIO on cell-cycle progression, GSK3α/β protein level was measured in C2C12 cells treated with BIO dissolved in GM medium (3 μM) or Vehicle (DMSO) in GM condition comparing their expression with C2C12 cells treated with DM (Fig. 2A). Results show that BIO is able to negatively affect the protein level of GSK3α/β (Fig. 2A) at 24 h and 48 h in C2C12 cells. Interestingly, the Vehicle was inactive at 24 and 48 h. Densitometric analysis are reported in Supplementary Information, Fig. S1B. Original gel blots are shown in Supplementary Information, Fig. S7A.

**Figure 3:**
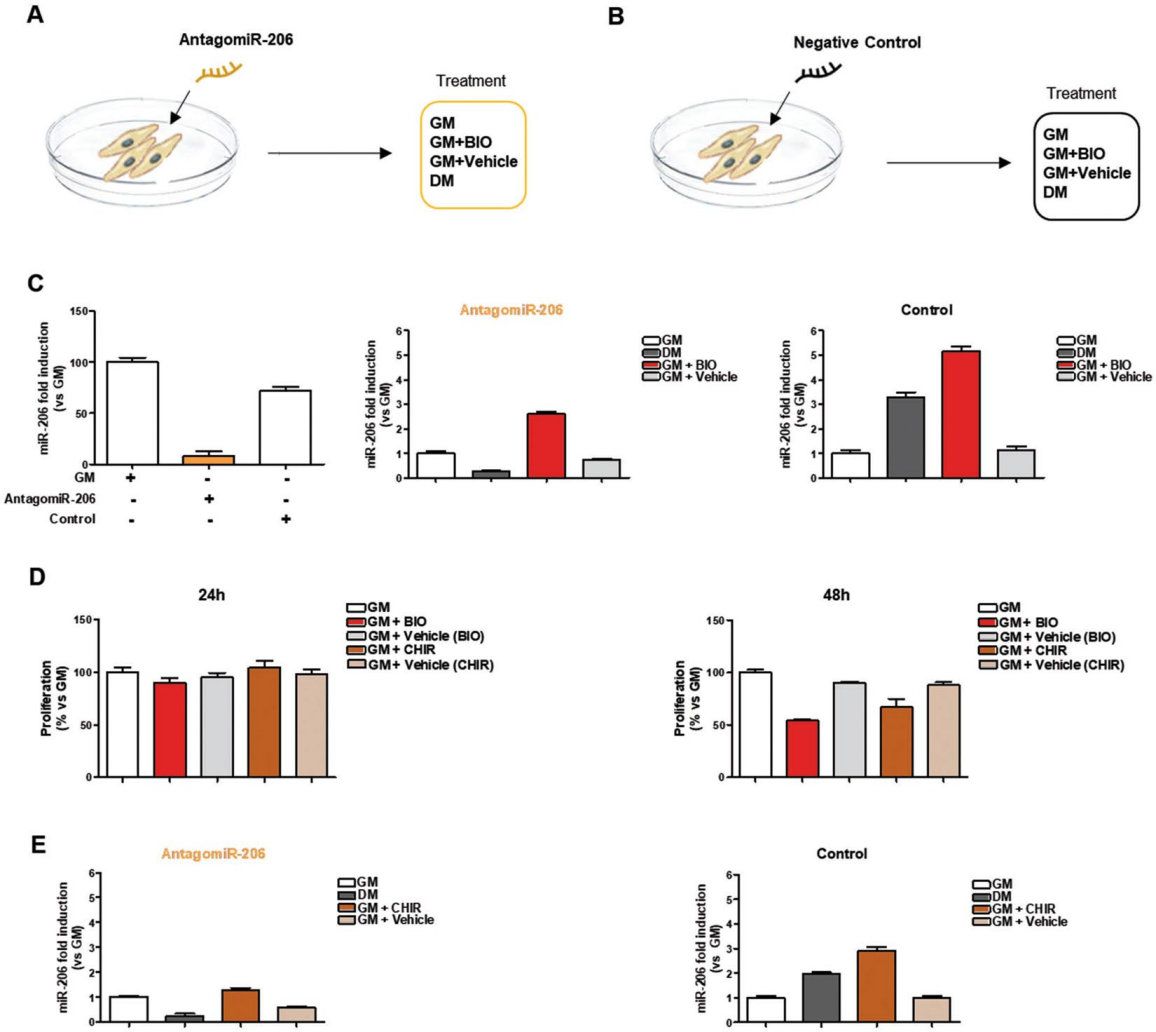
GSK3 inhibitors positively affects miR-206 expression. (**A,B**) Schematic representation C2C12 cells transfected with AntagomiR-206 or negative control treated with growth medium (GM), differentiation medium (DM) BIO (3 μM) or Vehicle (DMSO), for 24 h. (**C**) qRT-PCR analysis of miR-206 in C2C12 cells treated as described in panel A. Data (collected from three independent experiments) represent means ± SEM relative to GM treated cells. Data were analyzed by one-way analysis of variance ANOVA (***P < 0.001). (**D**) C2C12 cells were cultured in the presence of growth medium (GM), differentiation medium (DM), BIO (3 μM), Vehicle (DMSO), CHIR (3 μM) or Vehicle (DMSO), for 24 h and 48 h. The effect of CHIR on proliferation of C2C12 cells was measured by CellTiter-Glo® Assay. Data were collected from three independent experiments and represent means ± SEM relative to untreated cells (GM). Data were analyzed by one-way analysis of variance ANOVA: (**D**) left panel (ns), Fig. 1D right panel (**P < 0.01). (**E**) qRT-PCR analysis of miR-206 in C2C12 cells transfected with AntagomiR-206 or negative control treated with growth medium (GM), differentiation medium (DM), CHIR (3 μM) or Vehicle (DMSO), for 24 h. Data (collected from three independent experiments) represent means ± SEM relative to GM treated cells. Data were also analyzed by one-way analysis of variance ANOVA (***P < 0.001).

It has been shown that muscle differentiation relies on the exit from the cell cycle and this process is driven by many regulatory genes, including MyoD and Cyclin D1. It has largely been demonstrated that MyoD is a transcription factor that plays a key role in specifying myogenic progenitors during embryogenesis and preparing myoblasts for efficient differentiation^25^. Myoblasts expressing MyoD, withdraw from the cell cycle and then muscle-specific genes are activated^26^. Additionally, it has been shown that the absence of MyoD arrests myogenic stem cells in an intermediate proliferative stage that makes them unable to become full myogenic precursor cells^27^. CyclinD1 is the predominant cyclin that controls the rate of progression through the G1 phase of the cell cycle and it has been shown that repression of CyclinD1 is considered a hallmark of muscle cell differentiation^28–30^. Western blot analysis was performed on C2C12 cells treated with BIO dissolved in GM medium (3 μM) or Vehicle (DMSO) in GM condition comparing their expression with DM treatment only. Results show that BIO is able to reduce the protein level of Cyclin D1 (Fig. 2B) in C2C12 cells. Interestingly, the Vehicle was inactive at 24 and 48 h. Densitometric analysis are reported in Supplementary Information, Fig. S1B. Original gel blots are shown in Supplementary Information, Fig. S7B. Additionally, qRT-PCR assay was performed to evaluate CyclinD1 and MyoD gene expression level in C2C12-BIO in GM medium treated cells for 24 and 48 h. Results show a decrease of CyclinD1 and MyoD expression upon BIO treatment (Fig. 2C). MicroRNAs play a crucial role in regulating gene expression at post-transcriptional level. MiR-206 and miR-133 belong to a canonical muscle-specific microRNAs family (myomiRs) and play central regulatory roles in myoblast proliferation and differentiation. Particularly, an increase of miR-133 and miR-206 expression enhances myoblast proliferation and blocks cell cycle progression, respectively. Additionally, miR-206 induces myotube formation^24,31,32^. Thus, qRT-PCR analysis of C2C12-BIO treated cells at 24 and 48 h was performed to evaluate miR-133 and miR-206 gene expression. Results show an increased expression of miR-133 and miR-206 in C2C12-BIO treated cells in a time-dependent manner (Fig. 2D). Among the microRNAs involved in myogenesis, miR-27b has been shown to promote myogenic differentiation^33^. We investigated the effect of BIO on miR-27b expression: qRT-PCR analysis of C2C12-BIO treated cells at 24 and 48 h show an increased expression of miR-27b in a time-dependent manner upon BIO treatment, as shown in Fig. 2E. Since BIO has been shown to maintain self-renewal and pluripotency in human and mouse embryonic stem cells (ESCs) through Wnt/ β-Catenin signaling (also referred to as canonical Wnt signaling)^34,35^, Western blot analysis on C2C12-BIO treated cells at 24 and 48 h was performed in order to evaluate cytoplasmic/nuclear level of β-Catenin and results show an increased protein level of β-Catenin at 48 h. Conversely, C2C12-DM treated cells indicate no changes in β-catenin protein expression (Fig. 2F). Densitometric analysis are reported in Supplementary Information, Fig. S1C. Original gel blots are shown in the Supplementary Information, Fig. S7C. In addition, qRT-PCR on C2C12-BIO treated cells at 24 and 48 h and C2C12-DM treated was performed to evaluate β-catenin levels. Results show a decrease at 24 h and an increased expression of cytoplasmic/nuclear level at 48 h of β-Catenin in C2C12-BIO treated (Supplementary Information, Fig. S1D). In order to better investigate whether BIO, in our treatment condition, is able to activate Wnt ligands and induce disruption of the complex, Western blot analysis on C2C12-BIO treated cells at 24 and 48 h was performed. Nuclear level of β-Catenin was evaluated and results show an increased protein level of β-Catenin at 24 and 48 h. Conversely, C2C12-DM treated cells indicate no changes in β-catenin gene expression (Fig. 2G). Original gel blots are shown in Supplementary Information, Fig. S8. These results show that BIO, in our treatment condition, is able to inhibit GSK3 allowing the accumulation and nuclear import of β-catenin. Stabilization of the β-catenin protein plays a pivotal role in Wnt signaling pathway and the ubiquitin proteasome pathway plays pivotal roles in the degradation of the majority of eukaryotic proteins. It has been shown that the interaction of β-catenin with the E3 ubiquitin ligase receptor (βTrCP-1) provokes β-catenin ubiquitination and degradation^36^. To investigate the mechanism of β-catenin stabilization upon BIO treatment, we performed Western blot analysis to evaluate βTrCP-1 protein level. Results show downregulation of βTrCP-1 at 48 h (Supplementary Information, Fig. S2A). Original gel blots are shown in the Supplementary Information, Fig. S7D. To better investigate into miR-206 regulation by BIO, C2C12 cells were transfected with AntagomiR-206 or negative control in order to force miR-206 downregulation and observe if BIO is able to restore miR-206 expression, as shown in the schematic representation (Fig. 3A,B). QRT-PCR analysis was performed and results revealed that antagomiR-206 transfection strongly decrease miR-206 expression. No effect was shown by negative control transfection (Fig. 3C, left panel). Additionally, C2C12 were transfected with antagomiR-206 or negative control and then treated with GM, DM, BIO or Vehicle. QRT-PCR analysis revealed that the decreased expression of miR-206 was restored by BIO treatment, whereas no changes in miR-206 expression was shown in C2C12 DM-treated (Fig. 3C, middle panel). Conversely, C2C12 transfected with negative control show upregulated expression in C2C12-BIO and C2C12-DM treated (Fig. 3C, right panel). CHIR99021, as well as BIO, is a GSK3 inhibitor able to activate Wnt/β-catenin signalling by stabilising β-catenin and promoting self-renewal of stem cells (ESCs)^37,18^. Proliferation assay was performed in order to compare the effect of BIO and CHIR, then C2C12 cells were treated with BIO, CHIR or Vehicle for 24 and 48 h. Results revealed that CHIR-treated cells have similar effect of BIO-treated cells in the inhibition of C2C12 proliferation (Fig. 3D). Furthermore, qRT-PCR analysis was performed on C2C12 cells transfected with antagomiR-206 or control and then treated with CHIR, highlighted its capability, similarly to BIO, to upregulate miR-206 expression (Fig. 3E). Additionally, the action of BIO on miR-206 upregulation is more efficient compared to CHIR treatment. Our results indicate that BIO impairs myoblasts proliferation promoting upregulation of microRNAs positively involved in muscle differentiation through GSK3 inhibition.

Two Wnt pathways has been identified: the canonical (Wnt/β-catenin pathway) and the non-canonical pathway (β-catenin-independent pathway). The non-canonical Wnt pathway involves Calcium dependent transcription pathway Calcineurin/NFAT that contributes to the initial events of myogenic differentiation through an NFATc3-dependent mechanism^38,39^. Calcineurin initiates skeletal muscle differentiation by activating MEF2 and MyoD and its expression is strongly correlated to Utrophin that accumulates at the level of the neuromuscular junction where it seems to participate in the full differentiation of the postsynaptic membrane domain. C2C12 cells were treated with BIO and Vehicle for 24 and 48 h and qRT-PCR analysis was performed on Utrophin and Calcineurin, genes involved in IGF-1/Akt pathway^39^ and Calcineurin/NFAT signaling respectively. The results show a decreased expression of both genes at 24 and 48 h of treatment (Supplementary Information, Fig. S2B) and confirm that BIO does not activate the non- canonical Wnt pathway.

### A pulse of BIO improves skeletal muscle differentiation *in-vitro*

Skeletal muscle cells are derived from the differentiation of myoblasts into myotubes through a process known as myogenesis. The myogenesis process can be divided into two phases: the first is represented by the generation of early myotubes whereas the second is marked by the generation of multinucleated mature myotubes^5,40^. To generate multinuclear myotubes, myoblasts exit the cell cycle and fuse to form multi-nuclei fibers known as myotubes^41^. To be effective, this process requires the expression of muscle regulatory factors (MRFs), including MyoD, Myogenin, Myosin heavy chain II (MHC) and muscle creatine kinase^42,43^. Specifically, induction of muscle-specific genes like MHC is essential to detect terminal skeletal muscle differentiation^44^. To evaluate the generation of multinucleated mature myotubes and the expression of markers of skeletal muscle differentiation, C2C12 cells were grown in GM medium and then shifted to DM medium for 6 days and immunofluorescence analysis and qRT-PCR experiments were performed. Results clearly show the shift from proliferating myoblasts to early and mature myotubes (Supplementary Information, Fig. S3A). Since 24 h of treatment with 3 μM BIO dissolved in GM medium reduced the proliferation ability of C2C12 cells, we hypothesized that a pre-incubation (pulse) of BIO may have a functional role in skeletal muscle differentiation.

**Figure 4:**
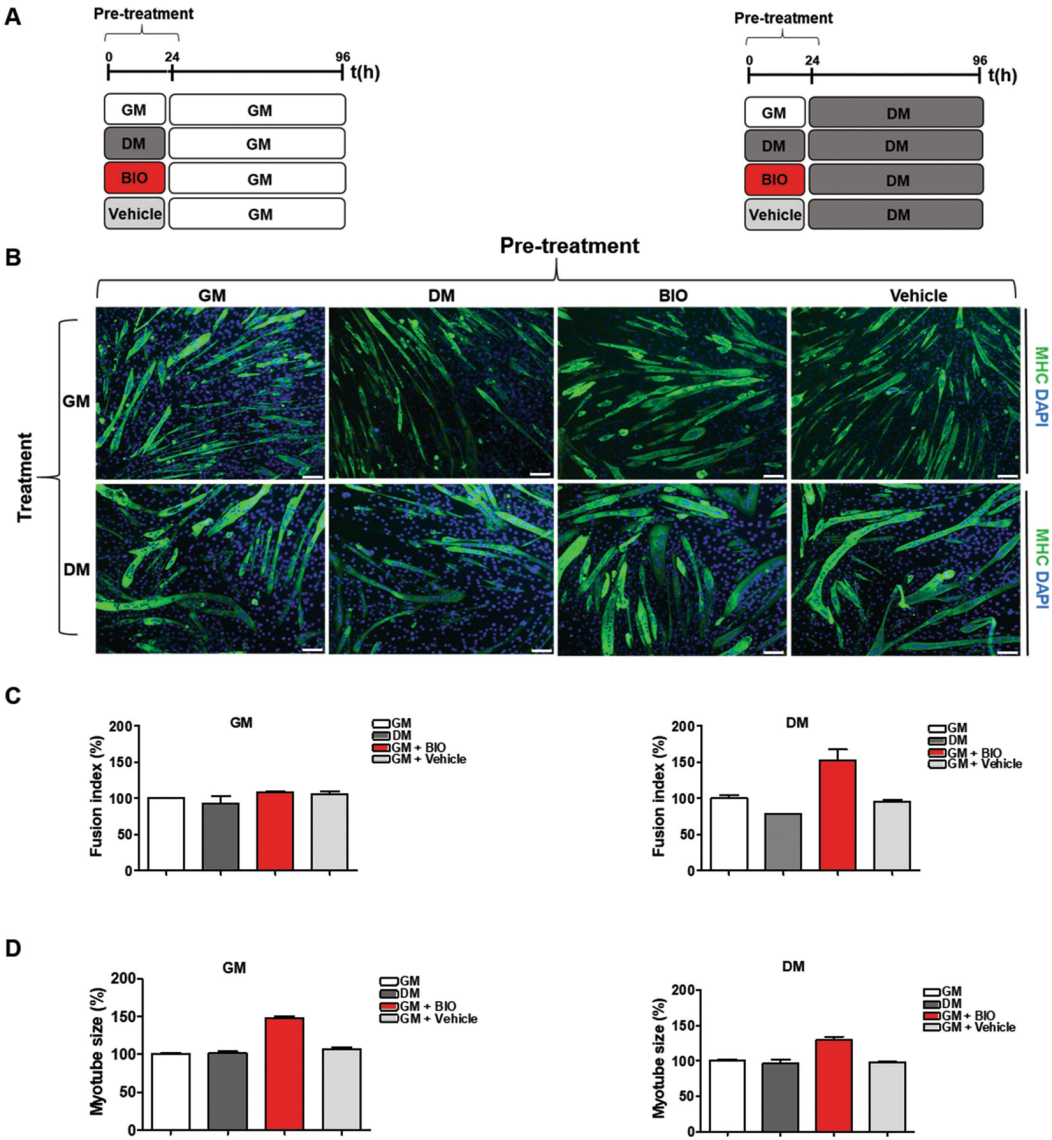
A pulse of BIO improves skeletal muscle differentiation *in-vitro*. (**A**) Schematic representation of C2C12 treatment: C2C12 cells were cultured in the presence of growth medium (GM), differentiation medium (DM), BIO (3 μM) or Vehicle (DMSO), for 24 h and then exposed to either GM or DM for 96 h. (**B**) Immunofluorescence analysis of differentiation marker MHC (green) in C2C12 cells. Nuclei are counterstained with DAPI dye (blue). (**C**) The histograms indicate the fusion index and the myotube size of all samples at day 4 of myogenic differentiation. The fusion index was calculated as the ratio of the number of nuclei inside myotubes (MHC positive cells) to the number of total nuclei at day 4 of myogenic differentiation. A myotube was defined by the presence of at least three nuclei within a continuous cell membrane. Data represent means ± SEM relative to GM or DM treated cells Data were analyzed by one-way analysis of variance ANOVA: (**C**) left panel (ns), (**C**) right panel (***P < 0.001). (**D**) Data of myotubes size are expressed as mean diameter in micrometers of ten myotubes measured per field. Bar scale = 100μm.Data (collected from four independent experiments) represent means ± SEM relative to GM or DM treated cells. Data were analyzed by one-way analysis of variance ANOVA (***P < 0.001).

Thus, to test whether BIO treatment improve myoblasts differentiation, C2C12 cells were cultured in GM, BIO dissolved in GM medium (3 μM), Vehicle (DMSO), or DM for 24 h (pulse) and then shifted to GM or DM for 6 days as schematized in (Fig. 4A). Immunofluorescence analysis was performed for α-MHC (green) detection and nuclei were stained with DAPI (blue) (Fig. 4B). Results revealed an increment of fusion index calculated on MHC-positive multinucleated myotubes compared to untreated (GM) or Vehicle (DMSO)-treated cells (Fig. 4C). In addition, together with the increase of fusion index, we also observed that the size of myotubes increased (Fig. 4D), consistent with skeletal muscle hypertrophy^45^. In addition, qRT-PCR of Utrophin and Calcineurin on C2C12 treated was performed as schematized in Fig. 4A and results show a decrease in expression level of Utrophin and Calcineurin genes involved in IGF-1/Akt pathway^39^ and Calcineurin/NFAT signaling respectively^46^ (Supplementary Information, Fig. S3B). However, it has been shown that Utrophin is a direct target of miR-206^47^ and that is able antagonize Calcineurin signaling during myotube hypertrophy^48^. Thus, the same experiments were performed for β-Catenin which expression has been shown decreased in mature myotubes (Supplementary Information, Fig. S3C). Taken together, these results revealed that BIO pre-treatment, and not DM or Vehicle, increased the fusion index and the size of myotubes highlighting a potential role of this compound in myogenic differentiation. To test whether CHIR99021 treatment improves myoblasts differentiation, as well as BIO, C2C12 cells were cultured in GM, DM BIO dissolved in GM medium (3 μM), Vehicle (DMSO), CHIR dissolved in GM medium (3 μM), or Vehicle (DMSO) for 24 h (pulse) and then shifted DM as schematized in Supplementary Information, Fig. S4A. Immunofluorescence analysis was performed for α-MHC antibody (green) and nuclei were stained with DAPI (blue) (Supplementary Information, Fig. S4B). Results show an increment of fusion index calculated on MHC-positive multinucleated myotubes compared to untreated (GM) cells (Supplementary Information, Fig. S4C). In addition, together with the increase of fusion index, we also observed an increase in the myotube size in C2C12-CHIR cells treated but not with the same extend of BIO.

### BIO promotes skeletal muscle differentiation in CTX-damaged myotubes *in-vitro*

**Figure 5:**
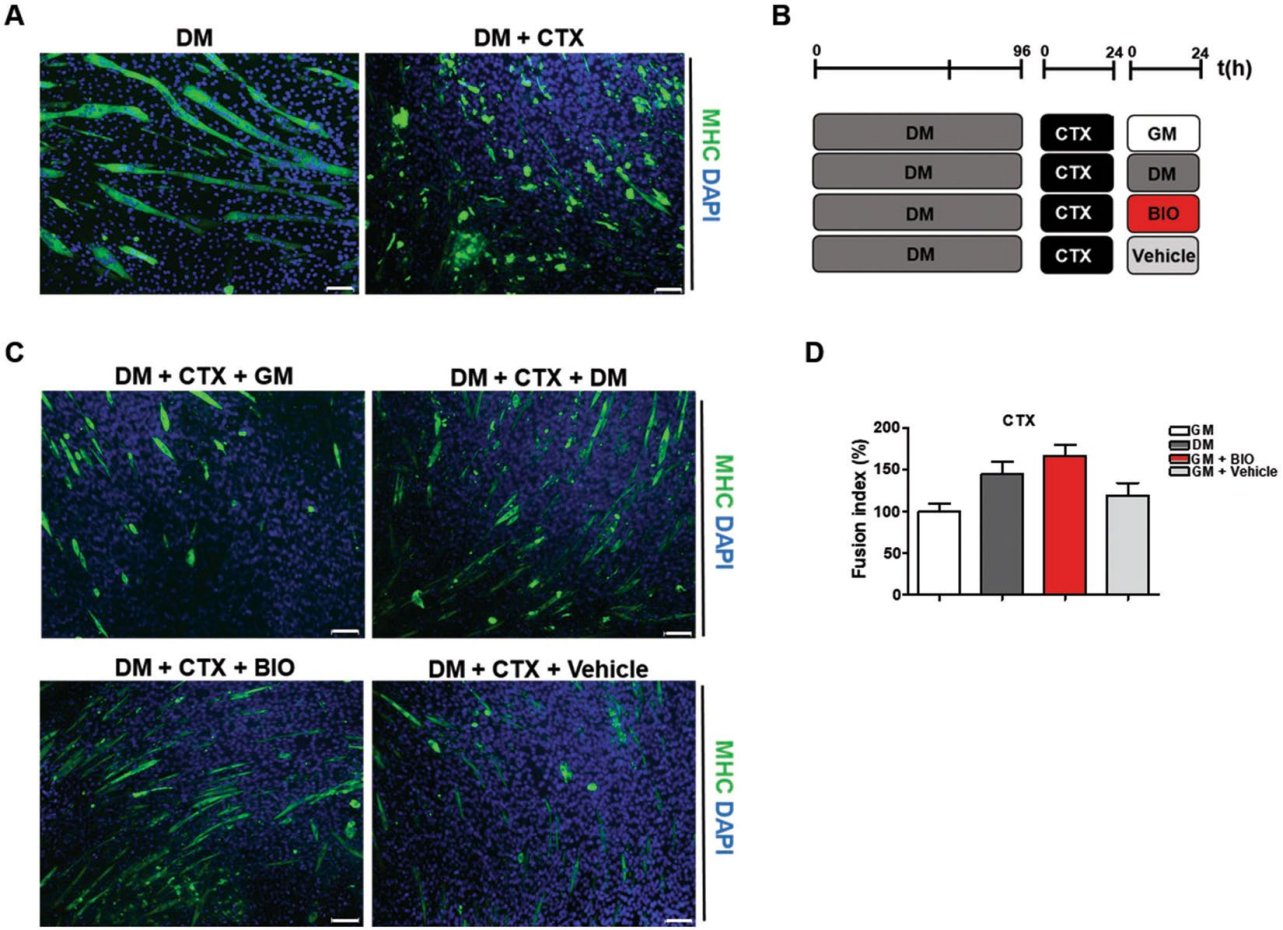
BIO promotes skeletal muscle differentiation in CTX-damaged myotubes *in-vitro* (**A**) Immunofluorescence analysis of myotubes stained for MHC detection (green) after exposure to differentiation medium (DM) alone or in combination with Cardiotoxin (CTX). Nuclei are counterstained with DAPI dye (blue) (**B**) Schematic representation of myotubes treatment: differentiated C2C12 cells (myotubes) were exposed to CTX (1 µM) for 24 h and to different stimuli, as represented in the experimental procedure, for 24 h. (**C**) Myotubes treated as described in B were subjected to immunofluorescence analysis for MHC (green). Nuclei are counterstained with DAPI dye (blue). Bar scale = 100 μm. (**D**) The histogram indicates the fusion index. The fusion index was calculated as the ratio of the number of nuclei inside myotubes (MHC positive cells) to the number of total nuclei at day 4 of myogenic differentiation. The number of nuclei was estimated by the average of nuclei counted in 15 independent and randomly chosen microscope fields. A myotube was defined by the presence of at least three nuclei within a continuous cell membrane. Data (collected from four independent experiments) represent means ± SEM relative to untreated cells (GM). Data were analyzed by one-way analysis of variance ANOVA (***P < 0.001).

To evaluate a potential role of BIO in muscle differentiation, we tested the effect of BIO treatment on damaged myotubes using a well-established protocol based on cardiotoxin (CTX) from Naja Mossambica injury as a model of chemically induced muscle damage^49,50^. Thus, C2C12 cells were differentiated *in-vitro* and the resulting myotubes were then incubated with or without CTX, which promotes myotubes disruption (Fig. 5A). Damaged myotubes were exposed to different stimuli for 24 h as schematized in Fig. 5B and then subjected to immunofluorescence analysis for MHC detection (Fig. 5C). Results revealed that BIO (dissolved in GM medium) counteracts CTX-induced damage more efficiently than DM or Vehicle (DMSO) as shown by the increased number of MHC-positive cells. Fusion index was calculated as reported in Fig. 5D. Collectively, our data indicate that BIO activates the response to injury in CTX-damaged myotubes inducing skeletal muscle differentiation. QRT-PCR analysis was performed on damaged myotubes treated with GM, DM, BIO or Vehicle (Fig. 5B) in order to detect Utrophin and Calcineurin expression. Results revealed a decreased level of Utrophin and no changes in the gene expression of Calcineurin in C2C12-CTX damaged cells exposed to BIO treatment, as well as, DM treatment (Supplementary Information, Fig. S5A). Transforming growth factor-β (TGF- β) regulates the skeletal muscle inflammatory response, inhibits muscle regeneration, regulates extracellular matrix remodeling, and promotes fibrosis^51^. Results show no changes in expression level of TGF- β in C2C12-CTX damaged cells exposed to BIO treatment, as well as GM and DM treatment (Supplementary Information, Fig. S5B). Myostatin is a member of the TGF- β family that negatively regulates skeletal muscle growth. It has been shown that its inhibition enhances muscle size and that it is a direct target of miR-206^24^. QRT-PCR analysis revealed a decreased expression of Myostatin in C2C12-CTX damaged cells exposed to BIO treatment, as well as, DM treatment (Supplementary Information, Fig. S5C). Additionally, the ubiquitin proteasome system is a proteolytic pathway essential in the maintenance of cellular homeostasis during differentiation and remodelling of skeletal muscle. Gene expression of E3 ubiquitin ligase complex has been investigated and qRT-PCR for MuRF-2, Cullin-1 and FBWX7 has been performed. MuRF-2 is dispensable for appropriate hypertrophic responses to hemodynamic stress *in-vivo*^52^, Cullin-1 is mainly involved in the terminal differentiation of the muscle^53^ and FBWX7 is a negative regulator of cell differentiation^54^. Results show no changes in gene expression (Supplementary Information, Fig. S5D–F). Taken together these results indicate that BIO treatment post damage helps cells to manage their regenerative capacity through the activation of the canonical Wnt/ β-catenin pathway but did not activate U3 Ubiquitin ligases to promote formation of new myotubes. In addition, no changes in β-TrcP expression responsible of β-catenin ubiquitination and proteasomal degradation^55^ was detected (Supplementary Information, Fig. S5G). Additionally, to confirm BIO action on GSK3, the same experiments were performed treating CTX-damaged cells with CHIR and results show similar effect of CHIR compared to BIO but conversely, the E3 ligase complex activation was occurred (Supplementary Information, Fig. S6).

### BIO positively regulates the expression of muscular markers and miR-206 *in-vivo*

**Figure 6:**
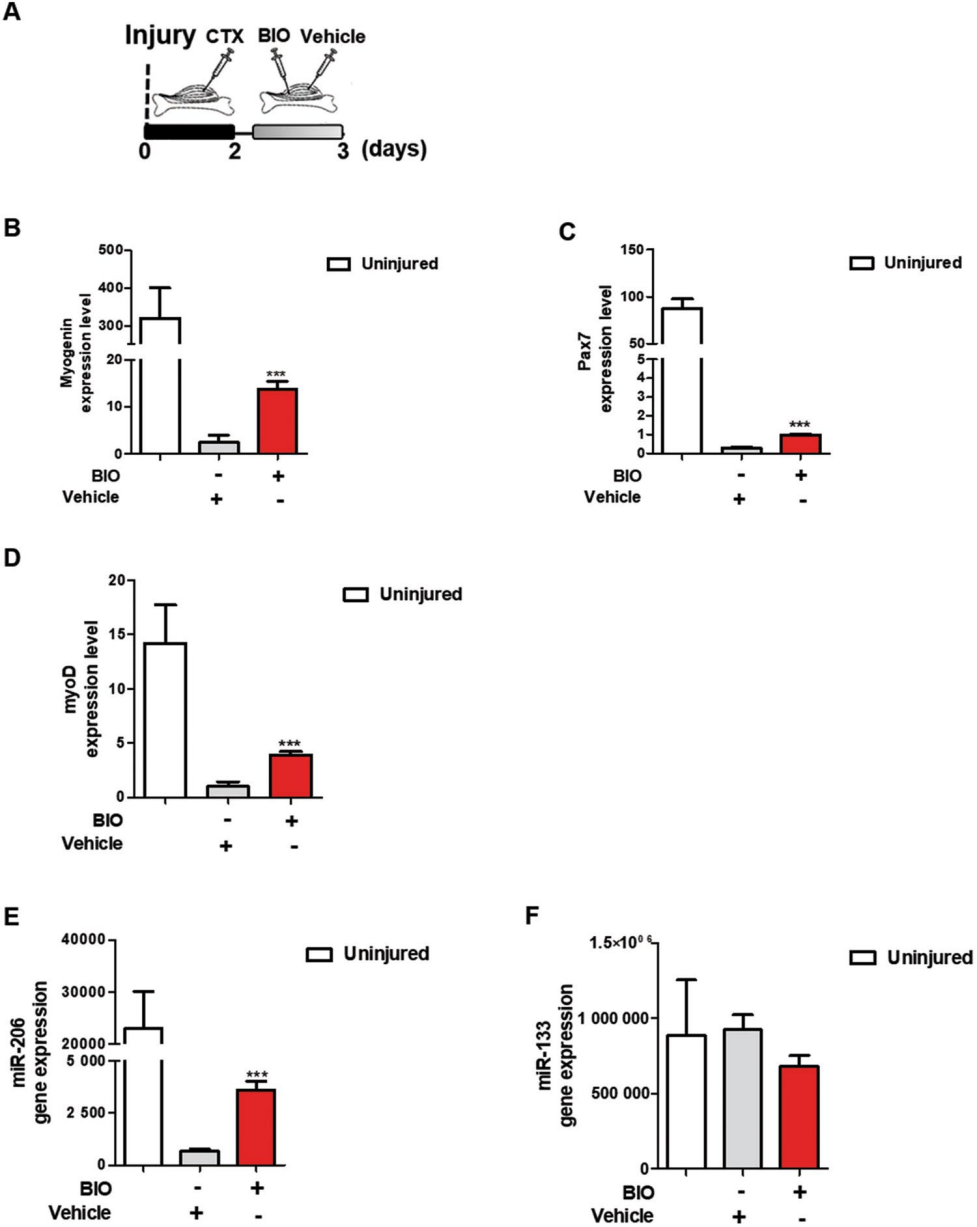
BIO positively regulates the expression of muscular markers and miR-206 *in-vivo*. (**A**) Experimental time-course of *in-vivo* muscle injury. Mice undergo direct muscle injections of BIO (1 μM) or Vehicle (PBS), as control, 2 days post-CTX injury and sacrificed after 24 h. QRT-PCR analyses of Myogenin (**B**), Pax7 (**C**) and MyoD (**D**) expression in TA muscle CTX-injured following 24 h of BIO treatment. Data are presented as the mean ± SEM. P < 0.05, **P < 0.01, ***P < 0.001 normalized to β-Tubulin (n = 5) according to two-tailed Student t test. QRT-PCR analyses of miR-206 (**E**) and miR-133 (**F**) expression in TA muscle CTX-injured following 24 h of BIO treatment. Results are normalized to U6 and miR-16 respectively. Data are presented as the mean ± SEM. P < 0.05, **P < 0.01, ***P < 0.001 (n = 5) relative to Vehicle treated cells according to two-tailed Student t test.

To further examine BIO’s effect in mice, murine Tibialis Anterior (TA) muscles were damaged with cardiotoxin (CTX) for three days and treated with BIO and Vehicle for 24 h as summarized in Fig. 6A. Muscle fibers were examined and qRT-PCR assay was performed. Results show an overall increase in Myogenin (Fig. 6B), Pax7 (Fig. 6C) and MyoD (Fig. 6D) expression. Additionally, miR-206 expression was strongly increased by BIO treatment (Fig. 6E); no effect was observed in miR-133 expression (Fig. 6F). These results suggest that BIO positively regulates the expression of muscular markers and that is able to increase miR-206 expression level during early myogenesis after muscular damage.

### BIO promotes differentiation in CTX-damaged TA muscle *in-vivo*

**Figure 7:**
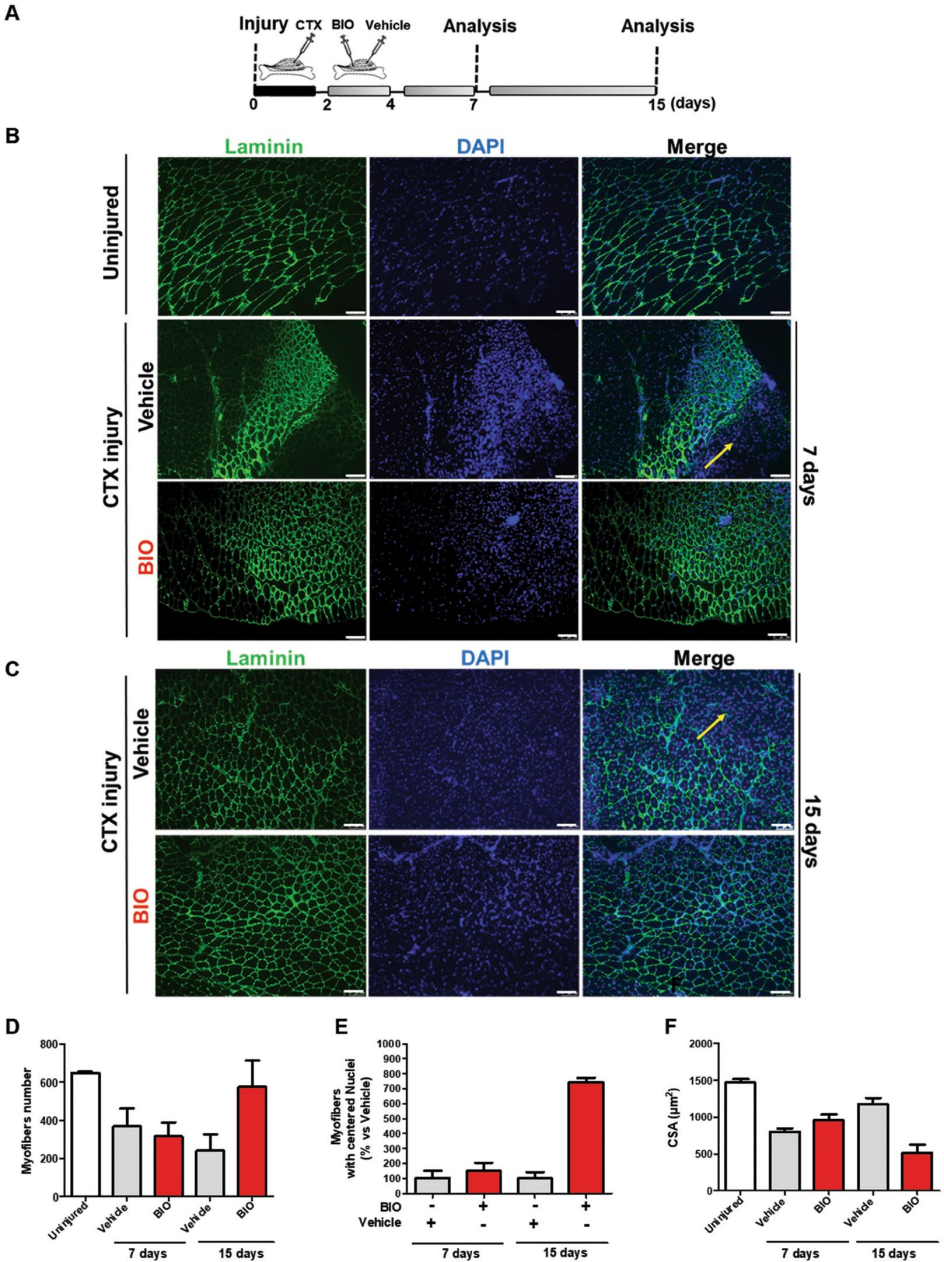
BIO affects nuclei position and myofibers number in CTX-damaged TA muscle *in-vivo*. (**A**) Experimental representation for *in-vivo* BIO treatment on TA muscle CTX-injured. Mice undergo direct muscle injections of BIO (1 μM) at 2 days and 4 days post-CTX injury and were sacrificed after 7 days and 15 days after CTX injection. (**B**) Representative cryosections of TA muscle CTX injured and treated with BIO and Vehicle at days 2 and 4. (Scale bars = 50 μm). Mice were sacrificed at 7days after CTX injection. Muscle were stained with Laminin and nuclei were counterstained with DAPI (blue). (**C**) Representative cryosections of TA muscle CTX injured and treated with BIO and Vehicle at days 2 and 4. (Scale bars = 50 μm). Mice were sacrificed at 15 days after CTX injection. Muscle were stained with Laminin^20^ and nuclei were counterstained with DAPI (blue). Bar scale = 100 μm. (**D**) Quantification of myofibers number in uninjured and CTX-injured TA muscles then treated with BIO or Vehicle. Data were analyzed by two-way analysis of variance ANOVA (***P < 0.001). (**E**) Myofibers with centralized nuclei of CTX-injured and treated with BIO or Vehicle and (**F**) CSA analysis of uninjured fibers and central nucleated fibers at 7 and 15 days following CTX injury and BIO treatment. The number of nuclei and myofibers with centralized nuclei was estimated by the average of 30 independent and randomly chosen microscope fields. CSA analysis was estimated by the average area (μm^2^) chosen in the same sections for analysis (n = 4). Data were analyzed by two-way analysis of variance ANOVA (***P < 0.001).

**Figure 8:**
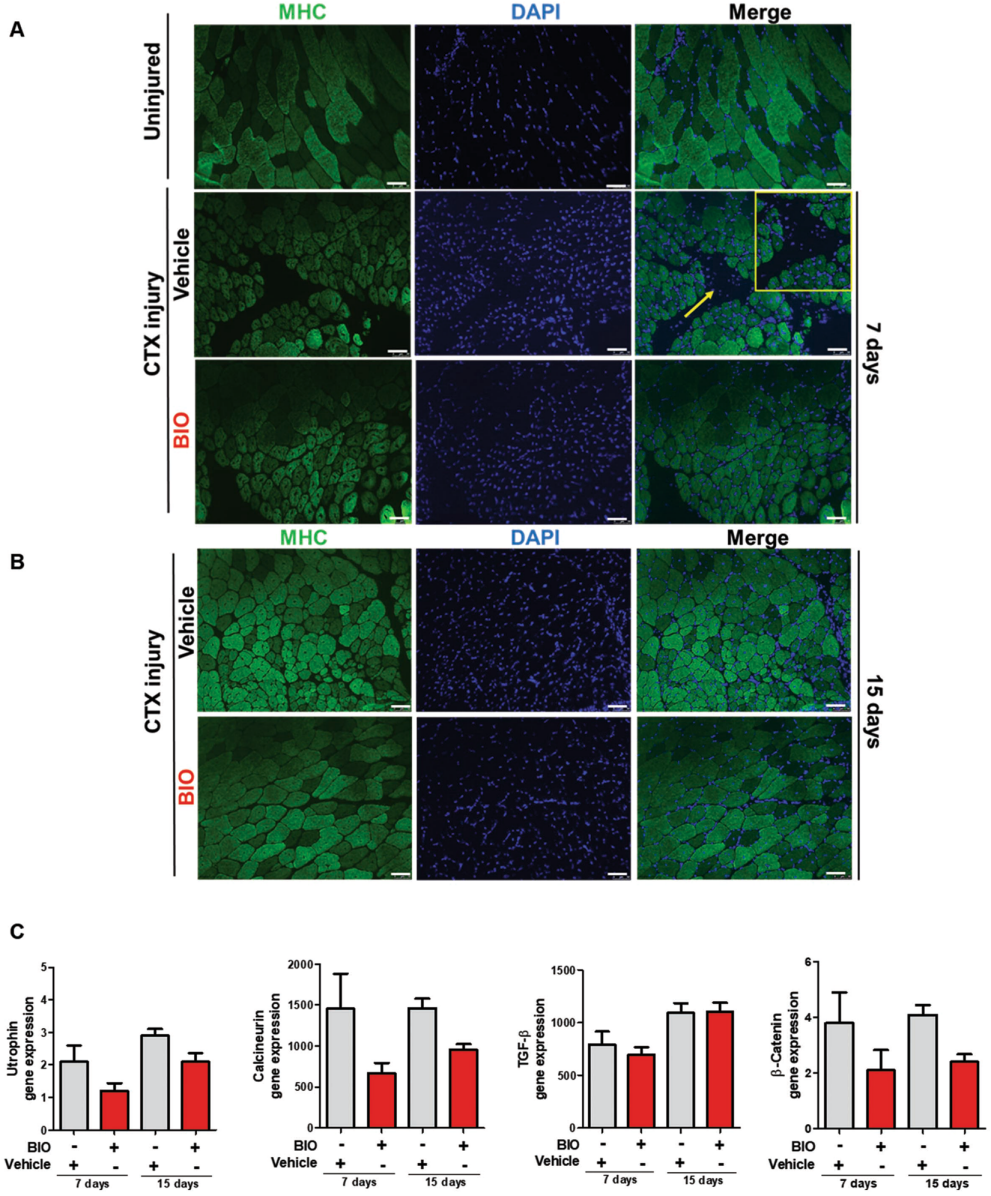
BIO promotes precocious differentiation in CTX-damaged TA muscle *in-vivo*. (**A**) Representative cryosections of TA muscle uninjured, CTX-injured and treated with BIO or Vehicle at days 2 and 4. (Scale bars = 50 μm). Mice were sacrificed at 7days after CTX injection. Muscle were stained with MHC antibody (green) and nuclei were counterstained with DAPI (blue). (**B**) Representative cryosections of TA muscle CTX-injured and treated with BIO and Vehicle at days 2 and 4. (Scale bars = 50 μm). Mice were sacrificed at 15 days after CTX injection. Muscle were stained with MHC antibody (green) and nuclei were counterstained with DAPI (blue). (**C**) qRT-PCR analysis of Utrophin, Calcineurin, TGF-β and β-Catenin expression level in TA muscle 7 days and 15 days in CTX-injured and treated with BIO or Vehicle normalized to β-Tubulin. Data are presented as the mean ± SEM (n = 4, P < 0.05, **P < 0.01, ***P < 0.001) relative to Vehicle treated cells. Data were analyzed by two-way ANOVA: Utrophin (*P < 0.05), Calcineurin (*P < 0.05), TGF-β (**P < 0.01), β-Catenin (ns).

**Figure 9:**
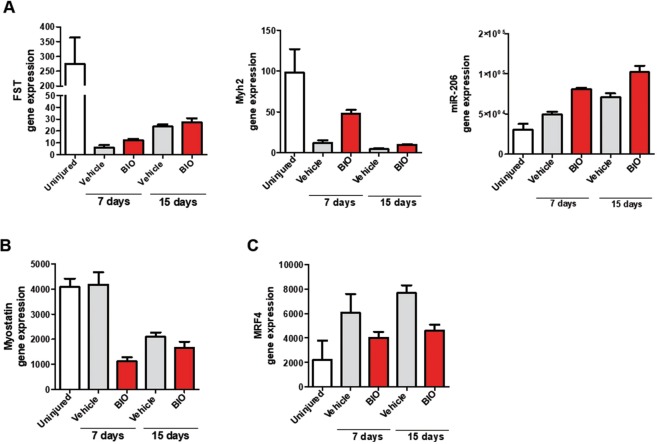
BIO’s effect on skeletal muscle markers and miR-206 in CTX-damaged TA muscle *in-vivo*. (**A**) QRT-PCR analyis of Follistatin (FST), Myh2 and miR-206 expression in TA muscle 7 days and 15 days after CTX injury and BIO or Vehicle treatment. Data are presented as the mean ± SEM (n = 4). Data were analyzed by two-way ANOVA: FST (***P < 0.001), Myh2 (*P < 0.05), miR-206 (*P < 0.05). Follistatin (FST) and Myh2 are normalized to β-Tubulin; miR-206 is normalized to U6. QRT-PCR analysis of Myostatin (**B**) and MRF4 (**C**) expression in TA muscle 7 days and 15 days after CTX injury and BIO or Vehicle treatment. Data are presented as the mean ± SEM (n = 4). Data were analyzed by two-way ANOVA: Myostatin (ns), MRF4 (*P < 0.05).

Skeletal muscles are composed of individual multinucleated myofibers with nuclei positioned at their periphery derived from fusion of mononucleated myoblasts. During their development, the position of the nuclei in myofibers is important for muscle function: during fusion of a myoblast into a myotube, the myoblast nucleus moves immediately to the center of the myotube as well as during muscle regeneration process and in some muscular diseases^56,57^.To better define if BIO treatment affects myofibers number and nuclei positioning, we treated TA muscle at 2 and 4 days following cardiotoxin (CTX) injury with either Vehicle (PBS) or BIO (Fig. 7A). At 7 and 15 days after injury the mice were sacrificed and the tissue analyzed. Immunofluorescence assay was performed to stain myofibers with α-Laminin antibody (green) and nuclei with DAPI (blue). Results revealed an altered muscle fibers morphology and nuclei positioning in BIO-treated TA muscle after 7 and 15 days post injury compared to Vehicle control (Fig. 7B,C). Furthermore, BIO treatment altered muscle fibers morphology in TA muscles clearly at 15 days after injury resulting in higher percentage of total myofibers and myofibers with centralized nuclei (Fig. 7D,E). Additionally, the mean CSA of central nucleated myofibers was significantly reduced providing a direct evidence for a role of BIO in skeletal muscle regeneration (Fig. 7F). These results indicate that BIO is able to promote the early regeneration process through initiating premature myogenic differentiation but also to delay it (as shown by the reduced CSA). Notably, BIO’s effect was analysed during the regeneration process, rather than at later time points when the regeneration process was almost completed and the myofibers fully regenerated. Interestingly, the increased differentiation observed at the 15 days of analysis is much more evident than at 7 days (BIO vs Vehicle).The same myofibers derived from TA muscle treated as in Fig. 7A–C were stained with MHC antibody (green) and nuclei with DAPI (blue) (Fig. 8A,B). MHC is required during embryonic and fetal myogenesis to regulate myogenic progenitor and myoblast differentiation. Additionally, myofibers in the non-injured muscles with peripheral nuclei and myofibers in the CTX-injured muscles with centralized nuclei as evidence of new myotube formation index were observed. QRT-PCR analysis was performed on RNA extracted from total TA muscle tissue in order to detect Utrophin, Calcineurin, TGF-β and β-Catenin expression. Results show an increase in the gene expression of Utrophin, Calcineurin, TGF-β genes in a time dependent manner. No changes in β-Catenin expression level was detected (Fig. 8C). Utrophin is similar in size and structure to dystrophin, helps to restore the linkage between the intracellular cytoskeleton and the extracellular matrix, thereby drastically decreasing the dystrophin associated disease pathology^58^. Calcineurin activation promotes the transcription of MEF2, Myogenin, MyoD and has been shown to control satellite cell differentiation, myofiber growth and maturation, all of which are involved in muscle regeneration^59^. Moreover, increased expression of TGF-β is able to promote development of fibrosis associated with wound healing and to participate in inflammatory response during muscle repair^60^. No changes in β-Catenin was detected (Fig. 8C). Additionally, we observed an overall increased muscle differentiation process as evidence of increased Follistatin (FST), Myh2 and miR-206 expression levels (Fig. 9A). Follistatin is a promising agent for improving skeletal muscle healing after injury and muscle diseases, such as the muscular dystrophies^61^. QRT-PCR was performed to analyze Myostatin level and results revealed a decreased expression compared to Vehicle, predominantly at 7 days of BIO treatment (Fig. 9B). Therefore, previous studies in the mdx mouse model of muscular dystrophy show that Myostatin inhibition attenuates several features of dystrophic muscle^62^. MRF4 is a negative regulator of muscle growth^63^ and its expression has been evaluated in CTX-injured muscles treated with BIO and Vehicle at 7 and 15 days. QRT-PCR was performed to analyze MRF4 level and results show a decreased in its expression predominantly at 15 days of BIO treatment compared to Vehicle (Fig. 9C). Taken together, these observations suggest the ability of BIO to affect nuclei position and myofibers number and its potential role in promoting regeneration process in CTX-damaged TA muscle *in-vivo*.

## Discussion

Glycogen synthase kinase-3 (GSK3) plays an important role in the regulation of several signaling pathways regulating key cellular processes such as cell cycle, inflammation and cell proliferation. Additionally, it has been associated with a broad spectrum of disease area: immunodeficiency syndrome (AIDS), malaria, inflammation, apoptosis, neuronal growth and canonical Wnt/β-catenin pathway. Treatment of embryonic stem cells (ESCs) with BIO results in increased β-catenin activity, activated canonical Wnt signaling and has been shown to promote self-renewal and pluripotency of stem cells. In growth conditions, canonical Wnt/β-catenin signaling primes myoblasts for myogenic differentiation and ectopic activation of canonical Wnt signaling *in-vivo* promoted premature differentiation during muscle regeneration following acute injury^64^. Based on these observations, we investigated into the role of BIO in the complex process of myogenesis using C2C12 as *in-vitro* model of myogenesis. We found that BIO negatively affects myoblasts proliferation promoting cell cycle exit to enhance the skeletal muscle differentiation process. Thus, we hypothesized a putative role of BIO in enhancing and promoting differentiation also in CTX-damaged myotubes *in-vitro*. Our results indicate a positive role of BIO in promoting newly formed myotubes in CTX-damaged myotubes *in-vitro*. Therefore, we confirmed its pro-differentiation effect after injury also by using a well-established chemotherapy agent able to interfere with DNA replication β-D-arabinofuranoside (Ara-C)^64–66^ as shown in Supplementary Information, Fig. S7E. It has been shown that skeletal muscle differentiation is associated with upregulation of skeletal muscle gene expression markers, increased myoblasts fusion into myotubes and increased myotube diameter. In addition, Jones *et al*.^67^, investigated the role of BIO in muscle regeneration demonstrating that BIO, as agonist of canonical Wnt signaling, is able to activate this pathway in satellite cells (muscle stem cells) during the core stages of muscle regeneration. Our i*n-vivo* investigations revealed that BIO alters muscle fibers morphology and nuclei positioning in CTX-injured TA muscles. In addition, CSA analysis on BIO-treated TA injured muscles revealed that BIO is not only able to increase skeletal muscle differentiation but is also able to modulate the regeneration process of damaged myofibers. It has recently been demonstrated that BIO is able to leads a slightly upregulation of the primary transcripts of miRNAs activated by Wnt/β-catenin signaling in stem cells^18^. Thus, we investigated a putative BIO modulation of a skeletal muscle-specific miRNA involved in muscle development, demonstrating the upregulation of miR-206 expression compared to canonical adult Horse Serum (HS). Several miRNAs are expressed in a tissue-specific manner highlighting a role of miRNAs in tissue-specific differentiation process. It has been reported that miR-206 represents a key modulator of skeletal muscle differentiation and has been identified as an important therapeutic target for the treatment of Duchenne Muscular Distrophy (DMD) by increasing Utrophin expression in skeletal muscles as a potential therapeutic approach for DMD. Recently, a study conducted by *Liu et al*. confirmed that miR-206 plays a protective role in muscular dystrophy: muscle injury leads to the activation of miR-206, which promotes the formation of new muscle fibers. They also demonstrated that the expression level of miR-206 decreased after the CTX injection, prior to gradually increase during the regeneration process induced by BIO^67^. Taken together, our studies suggest that the ability of BIO to increase miR-206 expression may exert a potential role in the myogenic differentiation process through the induction of miR-206 expression even if further investigations are needed to better define the functional relationship between BIO and miR-206 in the myogenic regeneration process.

## Supplementary information

Supplementary information

Table S1
