# Publisher Correction: 6-Bromoindirubin-3′-oxime intercepts GSK3 signaling to promote and enhance skeletal muscle differentiation affecting miR-206 expression in mice

**DOI:** 10.1038/s41598-020-66768-2

**Published:** 2020-06-19

**Authors:** Elvira Ragozzino, Mariarita Brancaccio, Antonella Di Costanzo, Francesco Scalabrì, Gennaro Andolfi, Luca G. Wanderlingh, Eduardo J. Patriarca, Gabriella Minchiotti, Sergio Altamura, Vincenzo Summa, Francesca Varrone

**Affiliations:** 1 IRBM S.p.A, 80131 Naples, Italy; 2 Department of Biology and Evolution of Marine Organisms, Stazione Zoologica Anton Dohrn, Naples, Italy; 3 Telethon Institute of Genetics and Medicine (TIGEM), 80078 Pozzuoli, NA Italy; 4 Stem Cell Fate Laboratory, Institute of Genetics and Biophysics, ‘A. Buzzati-Traverso’, CNR, 80131 Naples, Italy; 5 IRBM S.p.A, 00071 Pomezia, Italy; 6 Department of Pharmacy, University of Naples “Federico II”, 80131 Naples, Italy

Correction to: *Scientific Reports* 10.1038/s41598-019-54574-4, published online 02 December 2019

The original version of this Article contained errors.

Sergio Altamura was incorrectly listed as an equally contributing author in the original PDF version of the article. The original HTML version of the Article was correct.

Additionally, the original version of the Article did not disclose that Sergio Altamura was deceased at the time of publication. This information is now included.

Finally, the order of author names was incorrectly given as “Elvira Ragozzino, Mariarita Brancaccio, Antonella Di Costanzo, Francesco Scalabrì, Gennaro Andolfi, Luca G. Wanderlingh, Eduardo J. Patriarca, Gabriella Minchiotti, Sergio Altamura, Francesca Varrone & Vincenzo Summa”.

These errors have now been corrected in the HTML and PDF versions of the Article, and in the accompanying Supplementary Information files.

