# Supplementary material for: 6-Bromoindirubin-3′-oxime intercepts GSK3 signaling to promote and enhance skeletal muscle differentiation affecting miR-206 expression in mice
Source: Sci Rep. 2019 Dec 2;9:18091. doi: 10.1038/s41598-019-54574-4 (PMC6889408; doi:10.1038/s41598-019-54574-4)
Supplement: Supplementary file 1 — Supplementary information [file 41598_2019_54574_MOESM1_ESM.pdf]

**Supplementary Methods**

# 6-Bromoindirubin-3'-oxime intercepts GSK3 signaling to promote and enhance skeletal muscle differentiation affecting miR-206 expression in mice

Elvira Ragozzino[¶1], Mariarita Brancaccio[¶1,2], Antonella Di Costanzo[1], Francesco Scalabrì[1], Gennaro Andolfi[‡], Luca G. Wanderlingh[1¥] , Eduardo J. Patriarca[‡]., Gabriella Minchiotti[‡], Sergio Altamura[§†], Francesca Varrone[1*±] & Vincenzo Summa[§±]

**Count nuclei**

C2C12 cells, were plated in 96-well plates and incubated with GM, BIO (3 μM dissolved in GM medium) or Vehicle (DMSO) for 24 h and 48 h. Compounds were tested in triplicate on n=4 independent experiments. C2C12 treated cells were stained with Hoechst (33342, Thermo Fisher Scientific) and nuclei were counted by ImageJ software. Data are expressed as a percentage of control (GM). Data (collected from four independent experiments) represent means ± SEM. *P<0.05, **P<0.01, ***P<0.001 relative to GM stimulated cells (according to two-tailed Student t test).

**Quantitative real-time PCR**

RNA was extracted from cultured cells using TRIzol reagent (Ambion) followed by isopropanol-alcohol precipitation (RNeasy Mini Kit, Qiagen) before quantitation. RNA was then converted to cDNA with High Capacity cDNA Reverse Transcription kit (Applied Biosystem) according to the manufacturer's protocol. Gene expression analysis was carried out using the mouse housekeeping gene GAPDH. Primers for mouse Utrophin, Calcineurin, β-Catenin, Transforming growth factor beta (TGF-β), Myostatin, MuRF-2, Cullin-1, FBXW7 and β-TrCP are reported in **Supplementary Table S1**. Transcript levels were assessed using the Bio-Rad CFX machine according to the manufacturer's instructions and each experiment was repeated three times using independent RNA samples. Expression values of genes of interest were calculated based on ΔΔCt type of analysis normalizing on GAPDH reference gene. Data (collected from three independent experiments) represent means ± SEM. *P<0.05, **P<0.01, ***P<0.001 relative to GM stimulated cells (according to two-tailed Student t test).

**Immunoblotting and antibodies**

C2C12 cells were plated in 10cm plates and incubated with GM, BIO (3 μM dissolved in GM medium) or Vehicle (DMSO) for 24 h and 28 h. Total cell lysates were obtained with RIPA buffer supplemented with fresh 1X protease inhibitors (Complete Ultra Tablets Mini easypack, Roche) and phosphatase inhibitor cocktail tablets (phos STOP easypack, Roche). Commercial antibodies used in this study include anti-β-Catenin (Abcam), anti-GAPDH (Abcam) and anti-β-TrCP (Cell Signaling). Densitometric analysis of the bands was carried out using Image J software. Intensity of bands from the protein of interest were normalized to the intensity of GAPDH bands of the respective blots. Data are presented as the mean ± SEM (n = 2, P<0.05, **P<0.01, ***P<0.001), normalized to GAPDH.

**Mouse Skeletal Myoblasts treatments and analysis**

C2C12 cells (70% confluency) were treated 24 h with GM, DM, BIO dissolved in GM medium or Vehicle (DMSO) and then incubated in DM for 96 h. Gene expression analysis for Utrophin, Calcineurin and β-Catenin was carried out using the mouse housekeeping gene GAPDH. Transcript levels were assessed using the Bio-Rad CFX machine according to the manufacturer's instructions and each experiment was repeated three times using independent RNA samples. Data represent means ± SEM (n=4, *P<0.05, **P<0.01, ***P<0.001 relative to GM stimulated cells (according to two-tailed Student t test). C2C12 cells (70% confluency) were incubated in DM (96 h) and treated with Cardiotoxin (Sigma) (1 μM, 24

h). Subsequently the cells were treated 24 h with GM, DM, BIO 3 μM (dissolved in GM medium) or Vehicle. Gene expression analysis for Utrophin, Calcineurin, TGF-β, Myostatin, MuRF-2, Cullin-1, FBXW7 and β-TrCP was carried out using the mouse housekeeping gene GAPDH. Data represent means ± SEM (n=4, *P<0.05, **P<0.01, ***P<0.001 relative to untreated cells (GM) (according to two-tailed Student t test). To compare the effect of BIO and CHIR a on mouse skeletal myoblasts differentiation, C2C12 cells (70% confluency) were treated 24 h with GM, DM, BIO 3 μM (in GM medium), Vehicle (DMSO), CHIR 3 μM (in GM medium), or Vehicle (DMSO) and then incubated in DM for 96 h. The fusion index was calculated as described above. Data are expressed as mean diameter in micrometers of ten myotubes measured per field. The average size (diameter) per myotube was calculated as the mean of three measurements taken along the long axis of the myotube.

**Immunofluorescence**

C2C12 cells were plated in 96-well plates in GM medium and exposed in DM medium up to 96hours. After incubation treatment, cells were washed with 1X PBS (Gibco) and then fixed with 4% paraformaldehyde for 10 min. After three washes in PBS, cells were permeabilized with 0.5% Triton X-100 for 10 min and washed with PBS. Cells were then blocked with PBS 2% goat serum (Gibco) for 30 min and then incubated overnight at 4°C with anti-MHC antibody. Cells were then washed three times with PBS and incubated for 1 hour with Alexa Fluor 488-conjugated goat anti-mouse IgG (eBioscience) and after a washing step subjected to DAPI staining (Life Technologies). Cells were examined with a DMI600 (Leica) fluorescence microscope and photographed using a DFC360-FX camera (Leica). To test the effect of CHIR on mouse skeletal myoblasts differentiation, C2C12 cells were plated in 96-well plates in GM, DM, CHIR 3μM (dissolved in GM medium) or Vehicle (DMSO) and exposed in DM medium up to 96hours. After incubation treatment, cells were washed with 1X PBS (Gibco) and then fixed with 4% paraformaldehyde for 10 min and then stained as mentioned above.

**Mouse Skeletal Myocytes and Myotubes Ara-C treatment**

C2C12 cells (70% confluency) were incubated in DM for 96 hours and treated with 1 μM of Cardiotoxin (Sigma) for 24 hours. Subsequently the cells were treated with 50 μM of β-D-arabinofuranoside (Sigma) for 24 hours prior to incubate cells with 3 μM of BIO for 24 hours. To quantify the differentiation and fusion of C2C12 cells after treatments, samples were detected by α-MHC/DAPI staining and analyzed by ImageJ software in order to discriminate myotubes with a number of nuclei more than 5 (>5) and the total amount of $MHC^+$ cells.

**Supplementary Figures**

**6-Bromoindirubin-3'-oxime intercepts GSK3 signaling to promote and enhance skeletal muscle differentiation affecting miR-206 expression in mice**

Elvira Ragozzino[¶1], Mariarita Brancaccio[¶1,2], Antonella Di Costanzo[1], Francesco Scalabrì[1], Gennaro Andolfi[‡], Luca G. Wanderlingh[1¥] , Eduardo J. Patriarca[‡]., Gabriella Minchiotti[‡], Sergio Altamura[§†], Francesca Varrone[1*±] & Vincenzo Summa[§±]

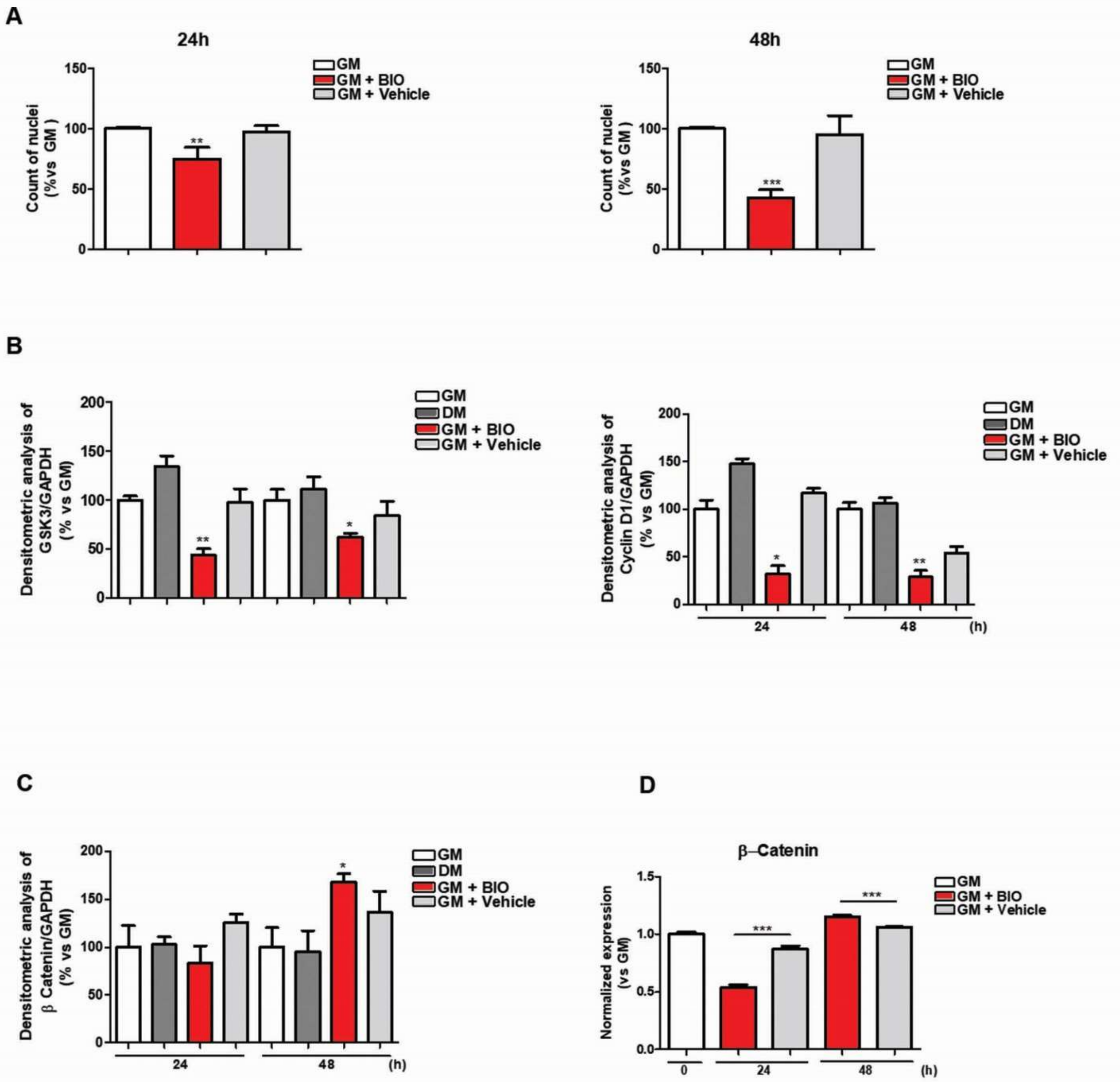
A
24h
48h
Count of nuclei (%vs GM)
GM
GM + BIO
GM + Vehicle
B
Densitometric analysis of GSK3/GAPDH (% vs GM)
Densitometric analysis of Cyclin D1/GAPDH (% vs GM)
GM
DM
GM + BIO
GM + Vehicle
24
48
(h)
C
Densitometric analysis of β Catenin/GAPDH (% vs GM)
D
β–Catenin
Normalized expression (vs GM)
0
24
48
(h)

**Figure S1**

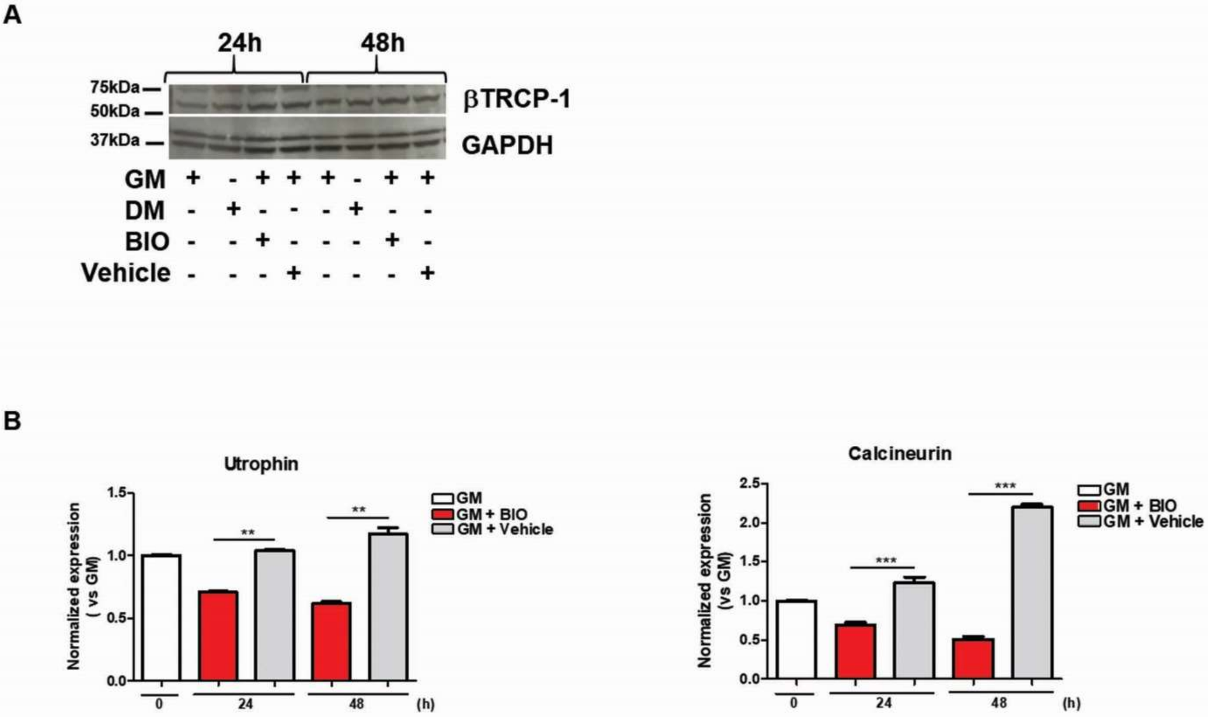
A
24h
48h
75kDa
50kDa
37kDa
βTRCP-1
GAPDH
GM + - + + + - + +
DM - + - - - + - -
BIO - - + - - - + -
Vehicle - - - + - - - +
B
Utrophin
Normalized expression ( vs GM)
GM
GM + BIO
GM + Vehicle
**
**
0 24 48 (h)
Calcineurin
Normalized expression (vs GM)
***
***
0 24 48 (h)

Figure S2

A

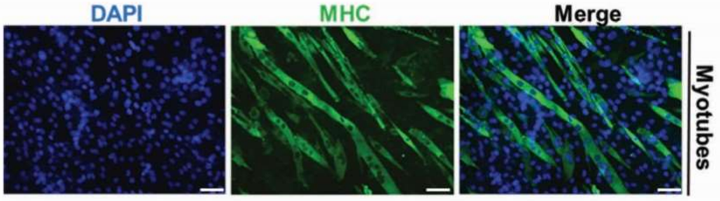


B

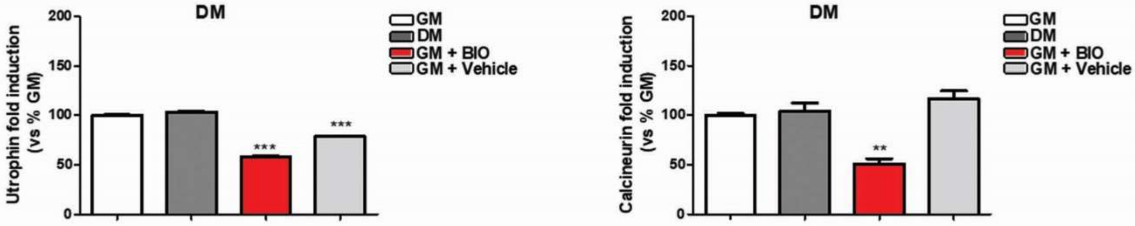


C

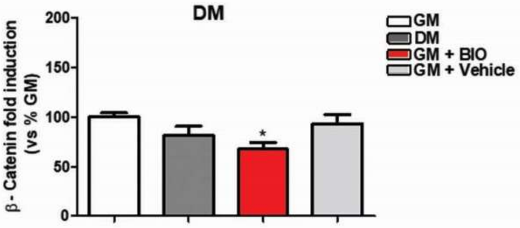


**Figure S3**

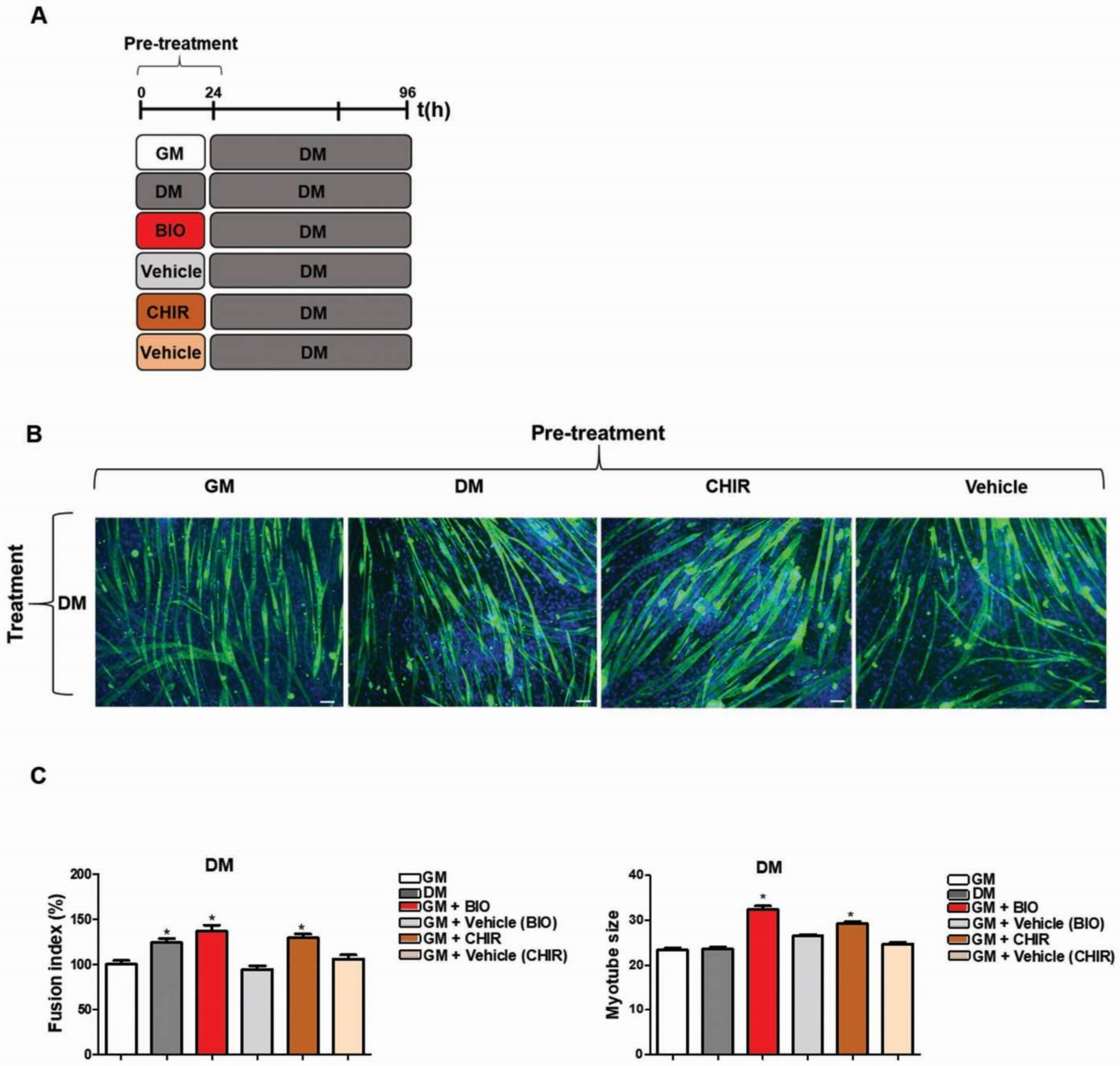
A
Pre-treatment
0
24
96
t(h)
GM
DM
BIO
Vehicle
CHIR
Vehicle
DM
B
Pre-treatment
GM
DM
CHIR
Vehicle
Treatment
DM
C
DM
Fusion index (%)
200
150
100
50
0
GM
DM
GM + BIO
GM + Vehicle (BIO)
GM + CHIR
GM + Vehicle (CHIR)
DM
Myotube size
40
30
20
10
0

**Figure S4**

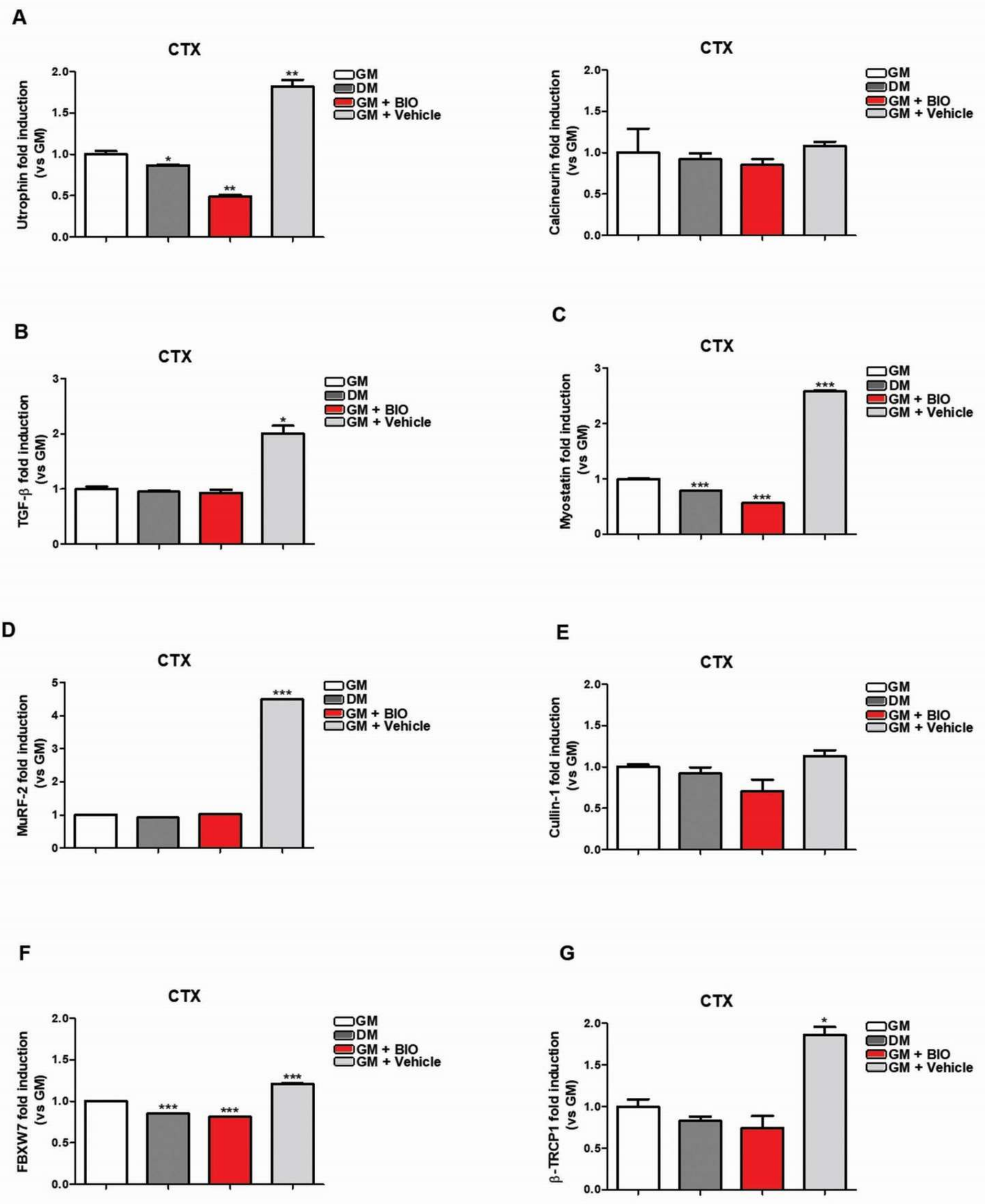

A
CTX
Utrophin fold induction (vs GM)
CTX
Calcineurin fold induction (vs GM)
B
CTX
TGF-β fold induction (vs GM)
C
CTX
Myostatin fold induction (vs GM)
D
CTX
MuRF-2 fold induction (vs GM)
E
CTX
Cullin-1 fold induction (vs GM)
F
CTX
FBXW7 fold induction (vs GM)
G
CTX
β-TRCP1 fold induction (vs GM)
GM
DM
GM + BIO
GM + Vehicle


**Figure S5**

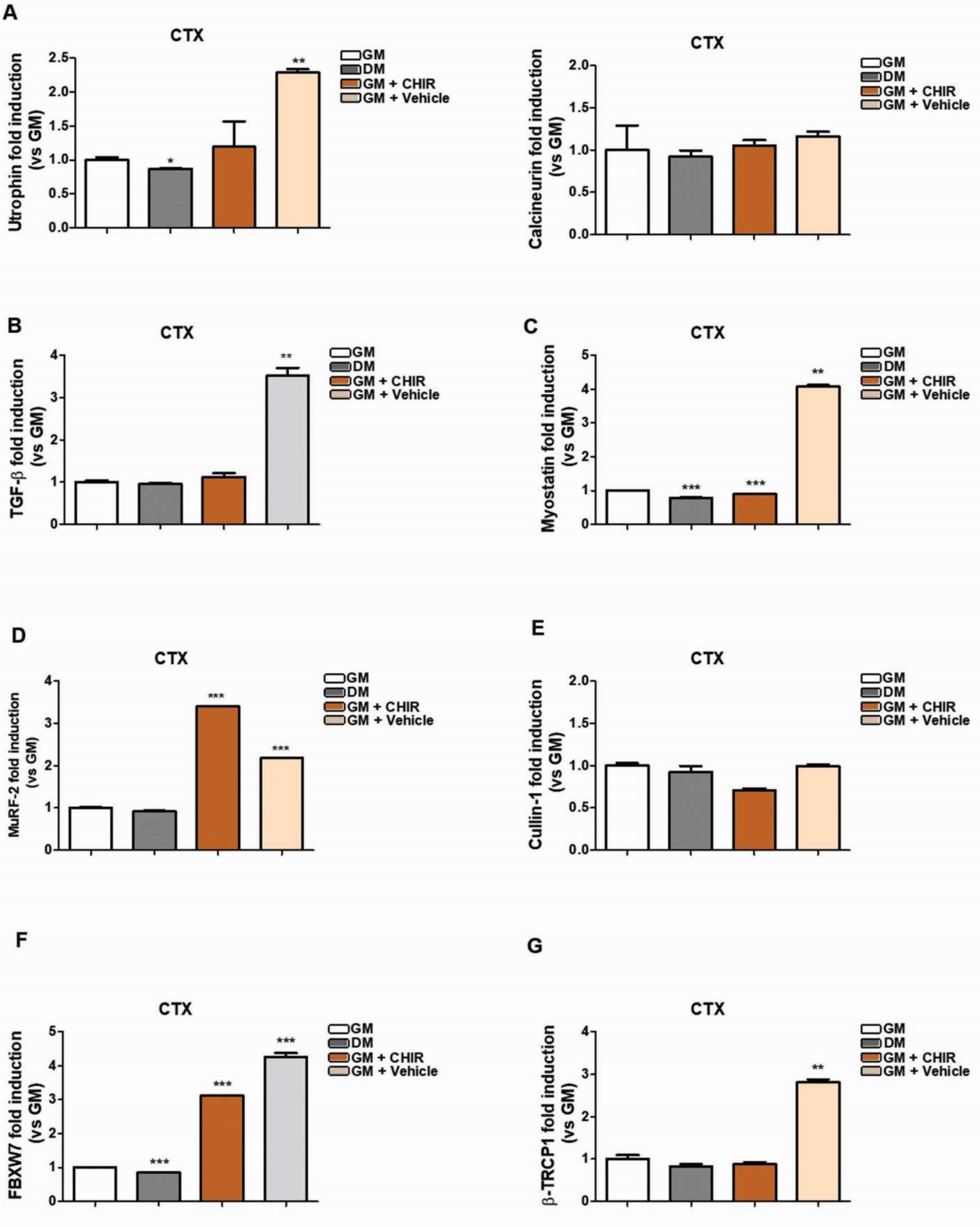
A
CTX
Utrophin fold induction (vs GM)
Calcineurin fold induction (vs GM)
B
TGF-β fold induction (vs GM)
C
Myostatin fold induction (vs GM)
D
MuRF-2 fold induction (vs GM)
E
Cullin-1 fold induction (vs GM)
F
FBXW7 fold induction (vs GM)
G
β-TRCP1 fold induction (vs GM)
GM
DM
GM + CHIR
GM + Vehicle
*
**
***

**Figure S6**

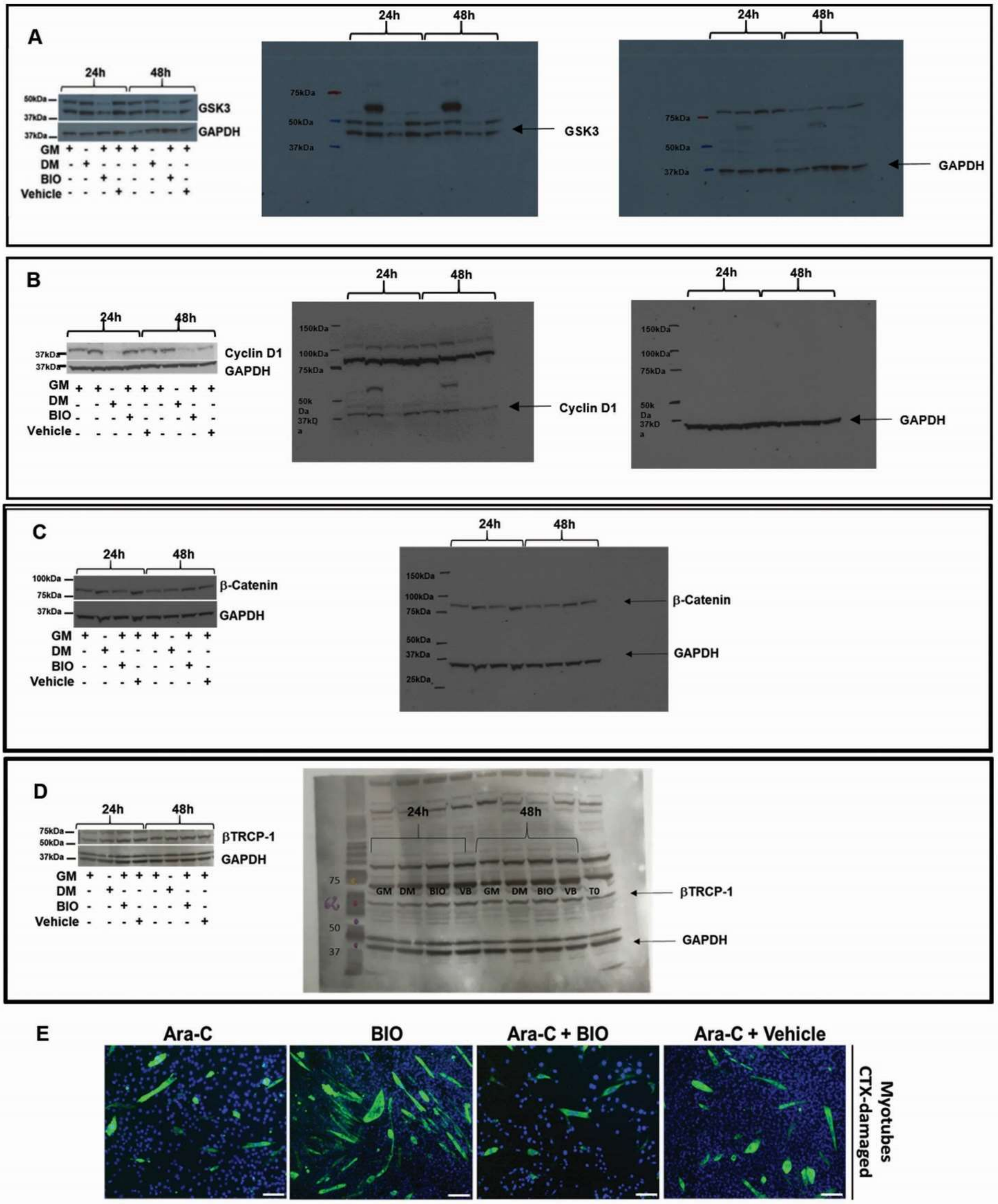
A
24h
48h
50kDa
37kDa
GSK3
GAPDH
GM + - + + + - + +
DM - + - - - + - -
BIO - - + - - - + -
Vehicle - - - + - - - +
75kDa
50kDa
37kDa
GSK3
GAPDH
B
Cyclin D1
GAPDH
150kDa
100kDa
75kDa
50k Da
37kD a
C
100kDa
75kDa
37kDa
β-Catenin
25kDa
D
βTRCP-1
GM
DM
BIO
VB
T0
75
50
37
E
Ara-C
BIO
Ara-C + BIO
Ara-C + Vehicle
CTX-damaged
Myotubes

Figure S7

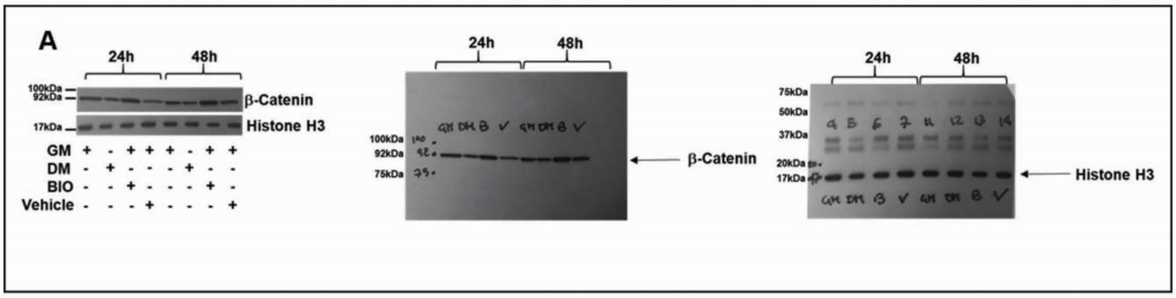
A
24h
48h
100kDa
92kDa
17kDa
β-Catenin
Histone H3
GM + - + + + - + +
DM - + - - - + - -
BIO - - + - - - + -
Vehicle - - - + - - - +
24h
48h
100kDa
92kDa
75kDa
β-Catenin
24h
48h
75kDa
50kDa
37kDa
20kDa
17kDa
Histone H3

Figure S8

**Supplementary Figure Legends**

**6-Bromoindirubin-3'-oxime intercepts GSK3 signaling to promote and enhance skeletal muscle differentiation affecting miR-206 expression in mice**

Elvira Ragozzino[¶1], Mariarita Brancaccio[¶1,2], Antonella Di Costanzo[1], Francesco Scalabrì[1], Gennaro Andolfi[‡], Luca G. Wanderlingh[1¥] , Eduardo J. Patriarca[‡]., Gabriella Minchiotti[‡], Sergio Altamura[§†], Francesca Varrone[1*±] & Vincenzo Summa[§±]

**Figure S1: (A)** BIO's effect on C2C12 nuclei measured by Hoechst assay. Data represent means ± SEM (n=2, *P<0.05, **P<0.01, ***P<0.001 relative to GM stimulated cells (according to two-tailed Student t test). **(B)** Densitometric analysis for data shown in the main figure **Fig. 2A** and **Fig. 2B** was carried out using Image J Software. Intensity of bands from the protein of interest were normalized to the intensity of GAPDH bands of the respective blots. Data represent means ± SEM (n=2, *P<0.05, **P<0.01, ***P<0.001 relative to GM stimulated cells (according to two-tailed Student t test). **(C)** Densitometric analysis for data shown in the figure **Fig. 2F** was carried out using Image J Software. Intensity of bands from the protein of interest were normalized to the intensity of GAPDH bands of the respective blots. Data represent means ± SEM (n=2, *P<0.05, **P<0.01, ***P<0.001 relative to Vehicle treated cells (according to two-tailed Student t test). **(D)** C2C12 cells were cultured in the presence of growth medium (GM), differentiation medium (DM), BIO (3 μM) or Vehicle (DMSO), for 24 h and 48 h. Cell lysates were subjected to Western blot analysis using anti-β-Catenin antibody. Data (collected from three independent experiments) represent means ± SEM. *P<0.05, **P<0.01, ***P<0.001 relative to Vehicle treated cells (according to two-tailed Student t test).

**Figure S2: (A)** C2C12 cells were cultured in the presence of growth medium (GM), differentiation medium (DM), BIO (3 μM) or Vehicle (DMSO), for 24 h and 48 h. Cell lysates were subjected to Western blot analysis of β–TrCP. **(B)** qRT-PCR analysis for Utrophin and Calcineurin was performed in C2C12 treated with GM, BIO (3 μM) or Vehicle (DMSO) for 24 h and 48 h. Data (collected from three independent experiments) represent means ± SEM. *P<0.05, **P<0.01, ***P<0.001 relative to Vehicle treated cells (according to two-tailed Student t test).

**Figure S3: (A)** Immunofluorescence analysis of C2C12 cells treated with DM medium: MHC protein expression (green) and nuclei counterstained by DAPI (blue) are shown. **(B, C)** qRT-PCR analysis for Utrophin, Calcineurin and β-Catenin was performed in C2C12 treated with GM, DM, BIO (3 μM) and Vehicle (DMSO) for 24 h and then shifted to DM medium for 6 days. Data (collected from three independent experiments) represent means ± SEM. *P<0.05, **P<0.01, ***P<0.001 relative to GM treated cells (according to two-tailed Student t test).

**Figure S4: (A)** Schematic representation of C2C12 treatment: C2C12 cells were treated with growth medium (GM), differentiation medium (DM), BIO (3 μM), Vehicle (DMSO), CHIR99021 (3 μM) or Vehicle (DMSO) for 24 h and then exposed to DM for 96 h. **(B)** α-MHC Immunofluorescence of myotubes treated with growth medium (GM), differentiation medium (DM), CHIR (3 μM) or Vehicle (DMSO) and then exposed to DM for 96 h. **(C)** C2C12 cells

treated as represented in Fig. S4A. Fusion Index and Myotube size of myotubes were calculated on MHC-positive cells. Data (collected from three independent experiments) represent means ± SEM. *P<0.05, **P<0.01, ***P<0.001 relative to GM treated cells (according to two-tailed Student t test).

**Figure S5:** C2C12 cells were incubated in DM medium for 96 h and treated with Cardiotoxin (Sigma) (1 μM, 24 h). Subsequently the cells were treated with GM, DM, BIO (3 μM) and Vehicle (DMSO). QRT-PCR analysis for **(A)** Utrophin, Calcineurin **(B)** TGF-β **(C)** Myostatin **(D)** MuRF-2 **(E)** Cullin-1 **(F)** FBXW7 and **(G)** β-TrCP was performed. Data are presented as the mean ± SEM (n = 3, P<0.05, **P<0.01, ***P<0.001), relative to GM treated cells (according to two-tailed Student t test).

**Figure S6:** C2C12 cells were incubated in DM medium for 96 h and treated with Cardiotoxin (Sigma) (1 μM, 24 h). Subsequently the cells were treated with GM, DM, CHIR (3 μM) and Vehicle (DMSO). QRT-PCR analysis for **(A)** Utrophin, Calcineurin **(B)** TGF-β **(C)** Myostatin **(D)** MuRF-2 **(E)** Cullin-1 **(F)** FBXW7 and **(G)** β-TrCP was performed. Data are presented as the mean ± SEM (n = 3, P<0.05, **P<0.01, ***P<0.001), relative to GM treated cells (according to two-tailed Student t test).

**Figure S7:** **(A)** Original gel blots for data shown in the main figure **Fig. 2A**. **(B)** Original gel blots for data shown in the main figure **Fig. 2B** **(C)** Original gel blots for data shown in the figure **Fig. 2F**. **(D)** Original gel blots for data shown in the figure **Fig. S2A**. **(E)** α-MHC Immunofluorescence of myotubes CTX damaged and treated with Ara-C +/- BIO and +/- Vehicle (DMSO).

**Figure S8:** **(A)** Original gel blots for data shown in the main figure Fig. 2G.
