# Supplementary material for: 6-Bromoindirubin-3′-oxime intercepts GSK3 signaling to promote and enhance skeletal muscle differentiation affecting miR-206 expression in mice
Source: Sci Rep. 2019 Dec 2;9:18091. doi: 10.1038/s41598-019-54574-4 (PMC6889408; doi:10.1038/s41598-019-54574-4)
Supplement: Supplementary file 2 — Table S1 [file 41598_2019_54574_MOESM2_ESM.pdf]

Supplementary Figure Tables

6-Bromoindirubin-3'-oxime intercepts GSK3 signaling to promote and enhance skeletal muscle differentiation affecting miR-206 expression in mice

Elvira Ragozzino[¶1], Mariarita Brancaccio[¶1,2], Antonella Di Costanzo[1], Francesco Scalabrì[1], Gennaro Andolfi[‡], Luca G. Wanderlingh[1¥] , Eduardo J. Patriarca[‡]., Gabriella Minchiotti[‡], Sergio Altamura[§†], Francesca Varrone[1*±] & Vincenzo Summa[§±]

Table S1: Oligonucleotide primers used in sample preparation for qRT-PCR

**CyclinD1**	**Fwd:** TCAAGTGTGACCCGGACTG	**Rev:** GGGGGAGAGGGCACATTAAG
**MyoD1**	**Fwd:** AGCACGCACACTTCTCTACT	**Rev:** GGCCTCATTCACTTTGCTCA
**β-Catenin**	**Fwd:** GTCTGAGGACAAGCCACAGGACTAC	**Rev:** AATGTCCAGTCCGAGATCA GCA
**Myogenin**	**Fwd:** TGAGTTCAGCAGCCATGAGT	**Rev:** CTCCGCCACAAAGACAGATG
**Pax 7**	**Fwd:** GAGCACTCGGCTAATCGAAC	**Rev:** CCGTGTTTCTCATGGTTGTG
**Utrophin**	**Fwd:** ACCAGCTGGACCGATGGA	**Rev:** CTCGTCCCAGTCGAAGAGATCT
**Calcineurin**	**Fwd:** AAGCTCTTTGAAGTGGGAGG	**Rev:** CATTTCCAAAGTCCTCCAGG
**TGF-β**	**Fwd:** TGCGCTTGCAGAGATTAAAA	**Rev:** CTGCCGTACAACTCCAGTGA
**FST**	**Fwd:** ACAAGAAGAATAAACCCCGCT	**Rev:** GTACTTCTAGTTCCGGCTGC
**Myh2**	**Fwd:** TGAGTTCAGCAGCCATGAGT	**Rev:** CTCCGCCACAAAGACAGATG
**Myostatin**	**Fwd:** CTCAGACCCGTCAAGACTCC	**Rev**: CTCTGCCAAATCAATACCAGTGCC
**MRF4**	**Fwd:** ATCAGCTACATTGAGCGTCTACA	**Rev:** CCTGGAATGATCCGAAACACTTG
**MuRF-2**	**Fwd:** CCGCTCGAGCCACCATGAGCACTTCTCTGAATTACAAG	**Rev:** TTTTCCCGGGCACCTTCATTTAGGGAATTCAACC
**Cullin-1**	**Fwd:** CAGCTCCACCATCAACATCAAC	**Rev:** AGTGCTGCTGGCATACACATT
**FBXW7**	**Fwd:** AAAGAGTTGTTAGCGGTTCTCG	**Rev:** CCACATGGATACCATC AAACTG
**β-TrCP1**	**Fwd:** CCCCAACTGACATTACCC	**Rev:** TCGAATACAACGCACCAA
**GAPDH**	**Fwd:** GGAGCCAAACGGGTCATCAT	**Rev:** TCACGCCACATCTTTCCAGA
